# Impact of the Gut Microbiota on Atorvastatin Mediated Effects on Blood Lipids

**DOI:** 10.3390/jcm9051596

**Published:** 2020-05-25

**Authors:** Friederike Zimmermann, Johann Roessler, David Schmidt, Andrzej Jasina, Paul Schumann, Martina Gast, Wolfgang Poller, David Leistner, Hector Giral, Nicolle Kränkel, Adelheid Kratzer, Sven Schuchardt, Markus M. Heimesaat, Ulf Landmesser, Arash Haghikia

**Affiliations:** 1 Department of Cardiology, Charité-Universitätsmedizin Berlin, Campus Benjamin Franklin, 12203 Berlin, Germany; Friederike.zimmermann@charite.de (F.Z.); Johann.roessler@charite.de (J.R.); david.schmidt@charite.de (D.S.); andrzej.jasina@charite.de (A.J.); paul.schumann@charite.de (P.S.); Martina.gast@charite.de (M.G.); Wolfgang.poller@charite.de (W.P.); david.leistner@charite.de (D.L.); hector.giral-arnal@charite.de (H.G.); Nicolle.kraenkel@charite.de (N.K.); adelheid.kratzer@charite.de (A.K.); ulf.landmesser@charite.de (U.L.); 2 DZHK (German Center for Cardiovascular Research), Partner Site Berlin, 10785 Berlin, Germany; 3 Berlin Institute of Health (BIH), 10178 Berlin, Germany; 4 Fraunhofer Institute for Toxicology and Experimental Medicine, Department of Bio and Environmental Analytics, 30625 Hannover, Germany; sven.schuchardt@item.fraunhofer.de; 5 Insitute of Microbiology, Infectious Diseases and Immunology, Charité-Universitätsmedizin Berlin, 12203 Berlin, Germany; Markus.heimesaat@charite.de

**Keywords:** gut microbiome, atorvastatin, cholesterol metabolism

## Abstract

Background and Aims: The mechanisms of interindividual variation of lipid regulation by statins, such as the low-density lipoprotein cholesterol (LDL) lowering effects, are not fully understood yet. Here, we used a gut microbiota depleted mouse model to investigate the relation between the gut microbiota and the regulatory property of atorvastatin on blood lipids. Methods: Mice (C57BL/6) with intact gut microbiota or antibiotic induced abiotic mice (ABS) were put on standard chow diet (SCD) or high fat diet (HFD) for six weeks. Atorvastatin (10 mg/kg body weight/day) or a control vehicle were applied per gavage for the last four weeks of dietary treatment. Blood lipids including total cholesterol, very low-density lipoprotein, low-density lipoprotein, high-density lipoprotein and sphingolipids were measured to probe microbiota-dependent effects of atorvastatin. The expression of genes involved in hepatic and intestinal cholesterol metabolism was analyzed with qRT-PCR. The alteration of the microbiota profile was examined using 16S rRNA qPCR in mice with intact gut microbiota. Results: HFD feeding significantly increased total blood cholesterol and LDL levels, as compared to SCD in both mice with intact and depleted gut microbiota. The cholesterol lowering effect of atorvastatin was significantly attenuated in mice with depleted gut microbiota. Moreover, we observed a global shift in the abundance of several sphingolipids upon atorvastatin treatment which was absent in gut microbiota depleted mice. The regulatory effect of atorvastatin on the expression of distinct hepatic and intestinal cholesterol-regulating genes, including *Ldlr, Srebp2* and *Npc1l1* was altered upon depletion of gut microbiota. In response to HFD feeding, the relative abundance of the bacterial phyla *Bacteroidetes* decreased, while the abundance of *Firmicutes* increased. The altered ratio between *Firmicutes* to *Bacteroidetes* was partly reversed in HFD fed mice treated with atorvastatin. Conclusions: Our findings support a regulatory impact of atorvastatin on the gut microbial profile and, in turn, demonstrate a crucial role of the gut microbiome for atorvastatin-related effects on blood lipids. These results provide novel insights into potential microbiota-dependent mechanisms of lipid regulation by statins, which may account for variable response to statin treatment.

## 1. Introduction

Statins are the most prescribed metabolic drugs for the treatment of patients with hypercholesterolemia with proven beneficial effects on patients’ prognosis both for primary [1] and secondary [2] prevention.

As inhibitors of 3-hydroxy-3-methyl-glutaryl-coenzyme A reductase (HMGCR) statins reduce the synthesis of cholesterol and increase the expression of low-density lipoprotein receptor (LDLR) resulting in decreased plasma LDL cholesterol (LDL) levels [3]. Accumulating evidence also suggests that statins mediate cholesterol removal via feces. In particular, atorvastatin mainly promotes cholesterol removal via the transintestinal cholesterol excretion pathway [4]. Although the modes of action of statins have been intensively investigated, there is still uncertainty about the mechanisms underlying interindividual variations in their regulatory property on plasma lipids, and in particular, their LDL lowering magnitude.

Increasing evidence implicate that metabolic drugs modulate both the composition and metabolic activity of the gut microbiota. This interaction is proposed to contribute to therapeutic effects of metabolic drugs, such as metformin [5], suggesting a relation between gut microbiota plasticity and individual response to pharmacotherapies. Thus, it is conceivable that the interindividual variability of therapeutic effects may depend on the composition of the gut microbiota, as well as microbe-related metabolites and their alteration upon treatment with subsequent regulatory effects on statin-mediated intestinal or hepatic regulation of cholesterol metabolism.

Here, we studied the influence of the gut microbiota on the modulatory effect of atorvastatin on the blood lipid profile in hypercholesterolemic mice with antibiotics-induced (ABS) depletion of the gut microbiota as compared to mice with intact gut microbiota (conventional, CONV). Additionally, the alteration of the gut microbiota composition was investigated upon treatment with atorvastatin.

The results of our study may shed further insight into the role of the gut microbiome for the lipid-regulating property of statins and help to provide novel gut microbiota related strategies to optimize the cholesterol-lowering effects of statins.

## 2. Methods

### 2.1. Mouse Experiments

**Figure 1:**
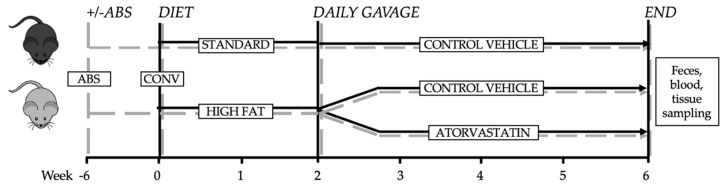
Experimental design. Secondary abiotic mice were generated by quintuple antibiotic treatment (+ABS). After pretreatment with or without antibiotics both ABS and CONV mice were exposed to either standard chow diet or high-fat diet. Two weeks after initiating the diet ABS and CONV mice were treated, either with control vehicle or atorvastatin for 4 weeks.

All animals were bred, raised and housed in the facilities of the “Forschungseinrichtungen für Experimentelle Medizin” (FEM, Charité—University Medicine Berlin, Germany) under specific pathogen-free (SPF) conditions with a 12 h-12 h day-night rhythm and unlimited access to food and water. Adult (16 weeks of age) C57BL/6J mice (Charles River) were placed on standard chow (SCD, Crude fat 4.1%, Cholesterol 14 mg/kg; E15000) or high-fat diet (HFD, Crude fat 34.6%, Cholesterol 290 mg/kg; E15741) (Ssniff, Soest, Germany) for 6 weeks prior to lipidome analysis. At the end of the 6 week-period mice were sacrified for collection of blood and organs (Figure 1). Mouse experiments were approved by the research advisory committee and permitted by LAGeSo (Landesamt für Gesundheit und Soziales Berlin, G0295/16).

### 2.2. Generation of Secondary Abiotic Mice

Secondary abiotic mice were generated through quintuple antibiotic treatment (+ABS) for 6 weeks via drinking water as shown previously [6]. In brief, mice were transferred to sterile cages and treated with a mix of ampicillin plus sulbactam (1 g/L), vancomycin (500 mg/L), ciprofloxacin (200 mg/L), imipenem (250 mg/L) and metronidazole (1 g/L) in autoclaved tap water (ad libitum). The intestinal colonization status of the mice was controlled once a week by highly sensitive cultural analysis of fecal samples. As early as three weeks of broad-spectrum antibiotic treatment, quality controls indicated virtually complete eradication of the intestinal microbiota, as demonstrated by negative results derived from both, culture and molecular detection of bacteria, using real-time PCR targeting of bacterial 16S rRNA genes. To avoid contaminations, mice were continuously kept in a sterile environment (autoclaved food and drinking water, sterile filtered antibiotic cocktail) and handled under strict aseptic conditions.

### 2.3. Molecular Analysis of Intestinal Microbiota

Major bacterial groups present in the murine gut microbiota were quantified by assessing the quantity of 16S rRNA gene copies per ng DNA applying quantitative real-time polymerase chain reaction with species-, genera- or group-specific 16S rRNA gene primers (Tib MolBiol, Berlin, Germany) (Supplementary Table S1).

Briefly, fecal samples were collected and preserved for further analysis with nitrogen. For DNA extraction the samples were suspended in PBS and centrifuged (16.000× *g*/10 min/4 °C). The probes were resuspended in 0.5 mL lysis buffer (500 mM Tris (pH 9.0), 20 mM EDTA, 10 mM NaCl, 1% SDS) and incubated with proteinase K (2 mg/mL; Sigma-Aldrich, St. Louis, MO, USA) for 1 h at 56 °C. After bead beating, total DNA was isolated by phenol extraction and quantified by using Quant-iT PicoGreen reagent (Invitrogen^TM^, Carlsbad, CA, USA) and adjusted to 1 ng per µL [6,7].

### 2.4. Histology

Following sacrifice, organs were immediately embedded in tissue freezing medium (Leica) and frozen on dry ice. Tissues were stored at −80 °C until further use. Thin Sections (5 um) of the liver were prepared on glass slides (Thermo Scientific, Super frost PLUS) using the Cryostat Microtome (Microm HM 560). Tissues were then stained with Oil Red O and counterstained with haematoxylin following standard staining protocols. Images were analyzed with ImageJ Software. Small intestinal tissues were fixed in paraffin. Sections (1–2 µm) were then stained with a polyclonal rabbit antibody (Novus Biologicals, #NB400-127) for detection of Niemann-Pick type C1 Like-1 protein.

### 2.5. qRT-PCR and Western Blot

Total RNA from liver and ileum was isolated with TRIzol reagent (Invitrogen™; 15596026) following manufacturer’s instructions and quantified using absorbance measurements on a NanoDrop (Thermo Scientific™, Waltham, MA, USA; ND-2000). Enzymatic DNA digestion was performed on total RNA from both tissues using RNase-free DNase I, (1 U/µg; Thermo Scientific™; EN0251). Total RNA (1.5 µg) was used for the reverse transcription of hepatic RNA using the High-Capacity cDNA Reverse Transcription Kit (Applied Biosystems™, Foster City, CA, USA; 4368814) and 0.5 µg of total RNA for intestinal tissue using the High-Capacity RNA-to-cDNA Kit (Applied Biosystems™; 4388950). Relative mRNA expression of the hepatic (*Cyp7a1*, *Fxr*, *Hmgcr*, *Ldlr*, *Pcsk9*, *Srebp2*) and intestinal genes (*Abca1*, *Abcg5*, *Acat2*, *Asbt*, *Npc1l1*, *Srb1*) and *Gapdh* as reference gene was analyzed using the following TaqMan Gene Expression Assays as primers (Applied Biosystems™; 4351372) (Supplementary Table S2).

Relative expression (triple determination) was examined by TaqMan Gene Expression Master Mix (Applied Biosystems™; 4369542) following the manufacturer´s instructions.

Isolation of total cellular proteins and protein expression levels by Western blotting using SDS-Page were performed according to standard protocols [8]. Rabbit polyclonal anti-LDLR (1:500; Abcam, Cambridge, UK) and anti-SREBP2 antibodies (1:500; NovusBio, Littleton, CO, USA) were used as primary antibodies and equal protein loading was verified by reprobing the membrane with a mouse monoclonal anti-GAPDH antibody (1:10,000, Merck, Kenilworth, NJ, USA). As secondary antibodies polyclonal goat anti-mouse and anti-rabbit antibodies were used (1:10,000, SouthernBiotech, Birmingham, AL, USA).

### 2.6. Metabolite Profiling and Lipoprotein Separation

For Metabolite profiling, all plasma samples were shipped on dry ice and analyzed at the Fraunhofer Institute for Toxicology and Experimental Medicine (ITEM), Hannover, Germany, using a targeted metabolomics kit (MxP® Quant 500 kit: BIOCRATES Life Sciences AG, Innsbruck, Austria). This approach allows simultaneous absolute quantification of up to 630 metabolites covering 26 compound classes including 14 small molecule and 12 lipid classes using a combination of liquid chromatography (Agilent 1290 Infinity II LC, Santa Clara, CA, USA) and mass spectrometry (AB SCIEX 5500 QTrap™ mass spectrometer; AB SCIEX, Darmstadt, Germany). After normalization and pre-processing of the data, using MetIDQ™ software (Biocrates, Innsbruck, Austria) for peak integration and calculation of metabolite concentrations, 15 sphingolipids, distinct acylcarnitines and bile acids were employed for further investigation in the present study, whereas the unmentioned metabolites are documented in the supplemental Table S3.

Fast performance liquid chromatography (FPLC) was used for lipoprotein separation by means of two Superose 6 columns connected in series.

### 2.7. Statistical Analyses

Database management and statistical analyses were performed with PRISM version 8.2.0 (GraphPad Software Inc., San Diego, CA, USA) and IBM SPSS Statistics 25 (IBM, Armonk, NY, USA).

Grubbs’test was performed to identify and exclude outliers. Continuous data were subjected to the Kolmogorov–Smirnov- and Shapiro–Wilk-test to determine their distribution and were expressed as mean ± standard error of the mean (SEM). Comparison of means of normally distributed data was performed by independent *t*-test and Mann-Whitney U-test was used if data were not normally distributed. Significance was assumed at a two-sided *p*-value ≤ 0.05.

## 3. Results

### 3.1. Differences in Diet-Induced Weight Gain in Conventionally Raised and Abiotic Mice

**Figure 2:**
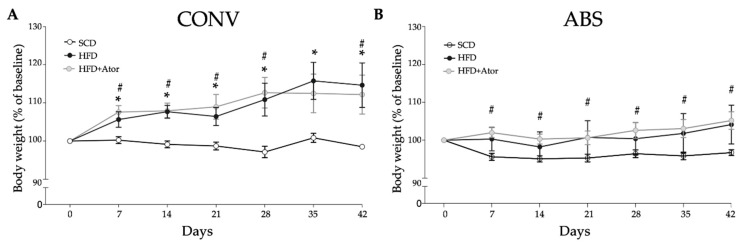
Course of body weight gain in CONV (**A**) (*n* = 15) and ABS (**B**) (*n* = 20) mice in response to SCD or HFD. Error bars indicate SEM.* = *p* <0.05 SCD vs. HFD, # = *p* < 0.05 SCD vs. HFD+Ator.

After diet initiation, weight change was monitored daily. As illustrated in Figure 2A, HFD induced significant weight gain in CONV mice (CONV+SCD vs. CONV+HFD: 98.5 ± 0.5 (% of baseline) vs. 114.7 ± 5.2 (% of baseline), *p* = 0.025), which was not affected by treatment with atorvastatin (CONV+HFD vs. CONV+HFD+Ator: 114.7 ± 5.2 (% of baseline) vs. 112.2 ± 4.6 (% of baseline), *p* = 0.76). Interestingly, this diet-induced weight gain was not observed in ABS mice (ABS+SCD vs. ABS+HFD: 97.1 ± 0.8 (% of baseline) versus 104.1 ± 4.6 (% of baseline), *p* = 0.08) (Figure 2B). This is in line with previously reported resistance to diet-induced obesity in germ-free mice [9]. In abiotic mice treatment with atorvastatin even significantly increased body weight (ABS+SCD vs. ABS+HFD+Ator: 97.1 ± 0.8 (% of baseline) vs. 105.2 ± 2.1 (% of baseline), *p* = 0.001) (Figure 2).

### 3.2. Effect of Atorvastatin on Cholesterol and Lipoprotein Levels in Conventionally Raised and Abiotic Mice

**Figure 3:**
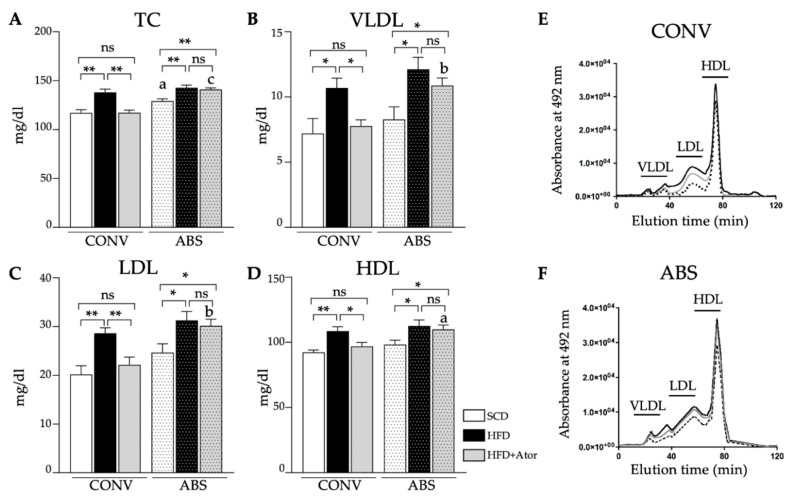

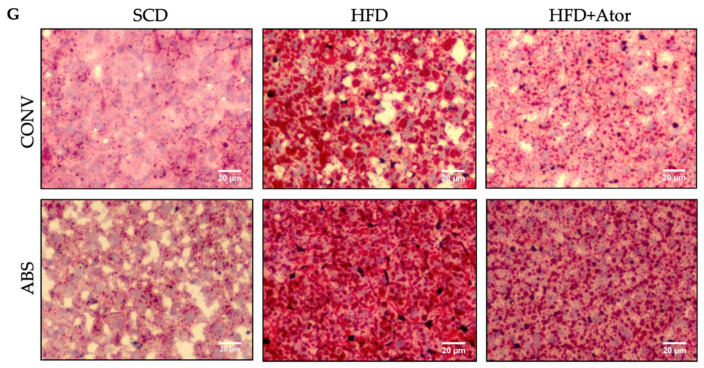
Analysis of plasma lipids. (**A**–**D**): Alterations of blood lipids in CONV (*n* = 14) and ABS (*n* = 13) mice under SCD or HFD and atorvastatin (Ator). (**E**,**F**): Representative HPLC plots of CONV (**E**) and ABS mice (**F**,**G**): Representative liver sections stained with hematoxylin-eosin and oil red o. Error bars indicate SEM. * = *p* < 0.05; ** = *p* < 0.01.

In CONV, as well as in ABS mice, HFD significantly increased serum levels of total cholesterol (TC) (Figure 3A–F). Similarly, levels of very low-density lipoprotein (VLDL), LDL and high-density lipoprotein (HDL) were significantly elevated upon HFD. While, treatment with atorvastatin significantly reduced levels of TC, VLDL, LDL and HDL in conventionally raised mice, it failed to show a significant effect on respective lipids in mice with depleted gut microbiota. Moreover, HFD induced hepatic fat deposition was reduced upon atorvastatin treatment in CONV mice while fatty liver damage in ABS mice remained high (Figure 3G).

### 3.3. Regulatory Effect of Atorvastatin on Blood Lipidome in Mice with Intact and Depleted Gut Microbiota under High Fat Diet

**Figure 4:**
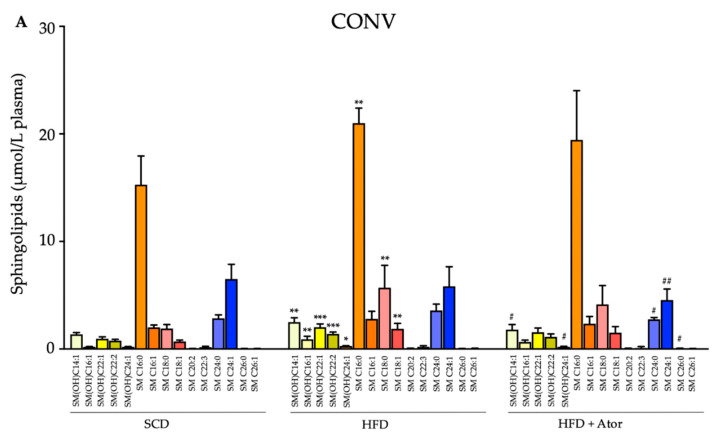

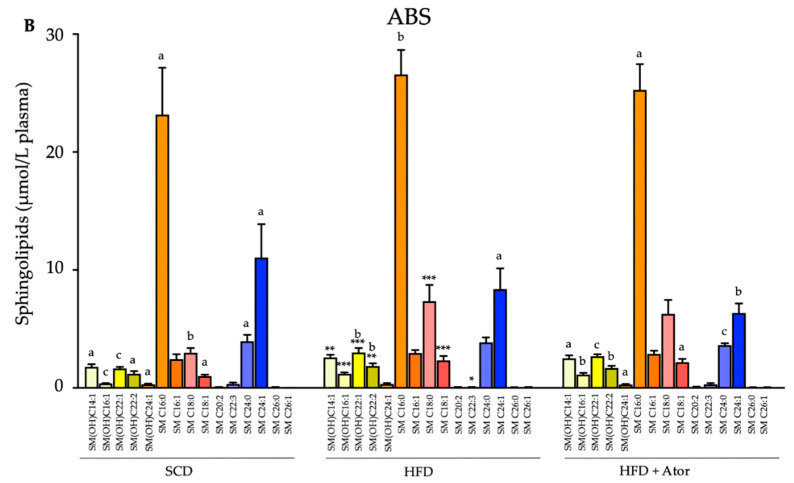
Blood sphingolipids in CONV (**A**) (*n* = 14) and ABS (**B**) (*n* = 13) mice under SCD or HFD and their changes upon Ator treatment. Error bars indicate SEM. Comparison between SCD vs. HFD is labelled with * and between HFD vs. HFD+Ator with #. The letters a,b and c indicate the comparison between CONV mice and the respective diet/treatment regimes in ABS mice. *, #, a indicate *p* < 0.05; **, ##, b indicate *p* < 0.01; ***, ###, c indicate *p* < 0.001.

To further investigate the impact of the gut microbiome on lipid-regulatory properties of atorvastatin we compared changes of a broad range of lipids including sphingolipids, ceramides, glycerophospholipids, triacylglycerols and glycosylceramides in HFD fed CONV and ABS mice with atorvastatin. The most striking difference between CONV and ABS mice was observed for sphingolipids. While, in both CONV and ABS mice the serum levels of many sphingolipids significantly increased upon HFD, treatment with atorvastatin prevented the increase in sphingolipid levels only in CONV mice, whereas sphingolipid levels remained largely unaffected upon atorvastatin treatment in ABS mice (Figure 4). Analyses of changes in the serum levels of glycerophospholipids, triacylglycerols, ceramides and glycosylceramides are provided in the supplementary table. As for these lipids, only minor differences were found between atorvastatin treated CONV-HFD and ABS-HFD mice (Table S3).

### 3.4. Atorvastatin Alters the of Gut Microbiota Composition

**Figure 5:**
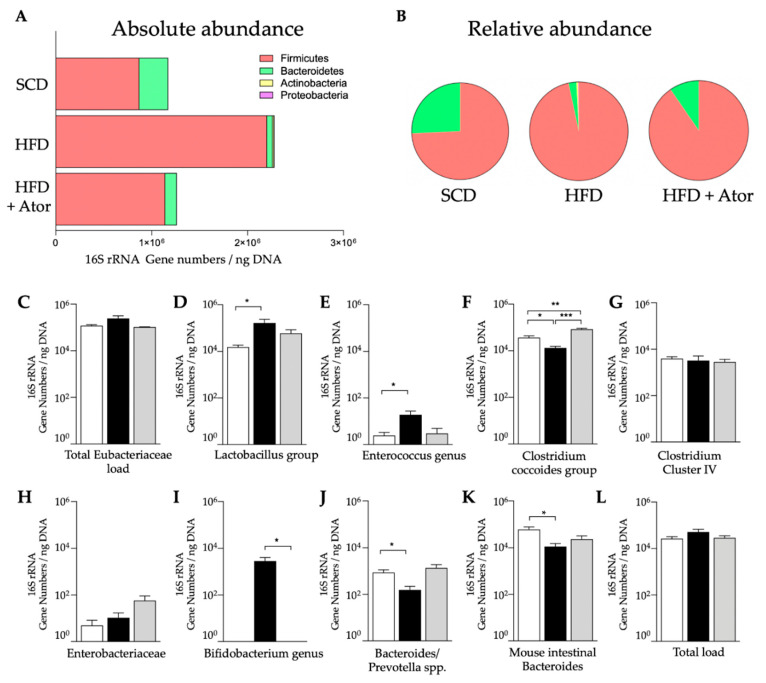
Analysis of intestinal microbiota. (**A**,**B**): Alterations of main intestinal bacterial phyla in response to HFD and the effects of Ator treatment (*n* = 15). (**C**–**L**): Analysis of 16S rRNA gene copies of distinct bacterial groups (*n* = 15). Error bars indicate SEM. * = *p* < 0.05, ** = *p* < 0.01; *** = *p* < 0.001.

In order to investigate how HFD alters the composition of gut microbiota and whether this alteration may be affected by treatment with atorvastatin, we collected fecal samples of all mice from the respective groups at the end of the diet and treatment period (Figure 1). As depicted in Figure 5, an increase in the absolute (A); and relative (B); abundance of phylum *Firmicutes* was found after HFD, as compared to SCD, while the absolute and relative abundance of bacterial phylum *Bacteroidetes* was decreased. Importantly, the increased ratio between *Firmicutes* to *Bacteroidetes* was partly reversed in HFD fed mice upon treatment with atorvastatin. Analysis of distinct bacterial groups displayed an increase in fecal gene copies of *Lactobacillus* group and *Enterococcus* genus by HFD, which was reversed to control levels upon treatment with atorvastatin (Figure 5D,E). Importantly *Bifidobaterium* genus was scarcely detected in SCD fed mice but increased in HFD. This augmentation was completely prevented in HFD fed mice treated with atorvastatin. *Clostridium coccoides* group was significantly decreased in mice upon HFD and increased even above control levels in response to treatment with atorvastatin.

### 3.5. Regulatory Effect of Atorvastatin on Hepatic and Intestinal Genes Involved in Cholesterol Metabolism in Mice with Intact and Depleted Gut Microbiome

**Figure 6:**
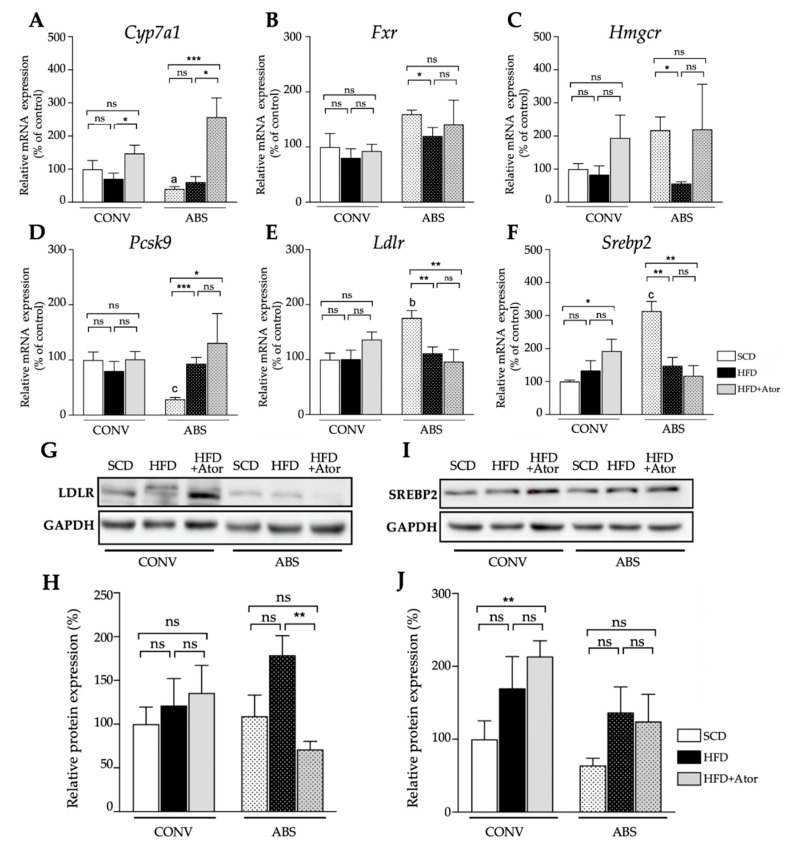
Hepatic genes and proteins. (**A**–**F**): Hepatic expression of genes involved in cholesterol metabolism and their alteration upon HFD + atorvastatin treatment in CONV (*n* = 15) and ABS (*n* = 19) mice. (**G**–**J**): Hepatic protein expression of sterol regulatory element-binding protein 2 (*Srebp*2) and ldl receptor (*Ldlr*) gene expression by atorvastatin in ABS (*n* = 12) mice as compared to CONV (*n* = 12) mice. The protein levels of srebp2 and ldlr were further evaluated by Western Blot (B). Error bars indicate SEM. * = *p* < 0.05, ** = *p* < 0.01; *** = *p* < 0.001.

**Figure 7:**
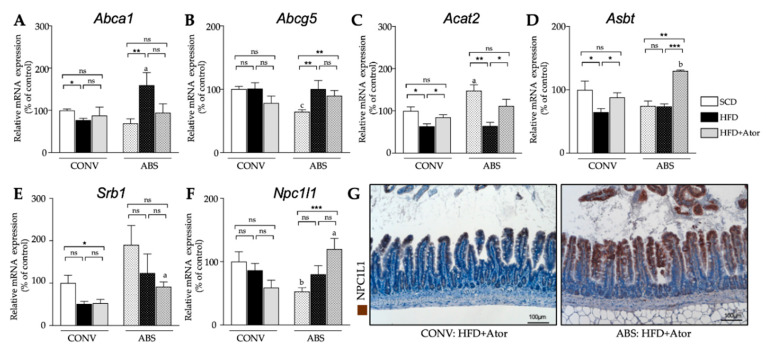
Intestinal gene and proteins. (**A**–**F**): Expression of cholesterol regulating genes in the small intestine and their alteration upon HFD and atorvastatin treatment in CONV (*n* = 15) and ABS (*n* = 20) mice. (**G**): Representative NPC1L1 stained histological sections of the ileum in CONV (left) and ABS (right) HFD fed mice under atorvastatin treatment. Error bars indicate SEM. * = *p* < 0.05, ** = *p* < 0.01; *** = *p* < 0.001.

**Figure 8:**
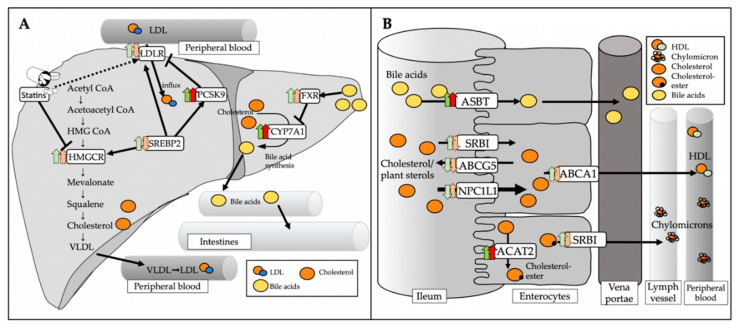
Role of key regulating genes involved in hepatic and intestinal cholesterol metabolism. (**A**): SREBP2 is a major transcription factor regulating intracellular cholesterol homeostasis. It activates the transcription of LDLR, HMGCR and PCSK9 to control cholesterol uptake by LDLR in a self-regulating circuit. CYP7a1 catalyzes the rate limiting step of hepatic bile acid synthesis. FXR, which is primarily activated by bile acids, regulates bile acid synthesis by CYP7a1 via a negative feedback loop. (**B**): NPC1L1 is the major transporter protein for intestinal cholesterol absorption. To a lesser extent, SRBI is also involved in cholesterol absorption and secretion into lymph. Free cholesterol is esterified by ACAT2 for further processing into chylomicrons. Excessive free cholesterol is secreted back into the intestinal lumen by ABCG5. ABCA1 mediates the efflux of cholesterol directly into the blood stream. ASBT is the major transporter protein for reabsorption of bile acids within the enterohepatic circulation. Green arrows indicate changes (dark green represents significant changes, bright green represents trends) in CONV HFD+Ator mice compared to CONV HFD mice. The red arrows indicate changes (dark red represents significant changes, bright red represents trends) in ABS HFD+Ator compared to ABS HFD mice.

Expression analysis of genes involved in cholesterol metabolism in the liver (Figure 6A) displayed a significant increase of mRNA levels of the bile acid-synthetic enzyme cholesterol 7α-hydrolase (*Cyp7a1*) in both CONV and ABS mice (CONV+HFD vs. CONV+HFD+Ator: 71.2 ± 16.6% vs. 147.2 ± 25.1%, *p* = 0.036; ABS+HFD vs. ABS+HFD+Ator: 60.7 ± 16.4% vs. 256.4 ± 58.0%, *p* = 0.012) indicating gut microbiota-independent regulatory property of atorvastatin on bile acid homeostasis. However, the mRNA expression of sterol regulatory element-binding protein 2 (*Srebp2*) (CONV+SCD vs. CONV+HFD+Ator: 100.0 ± 5.0% vs. 192.2 ± 36.4%, *p* = 0.037; ABS+SCD vs. ABS+HFD+Ator: 313.6 ± 29.3% vs. 117.6 ± 30.6%, *p* = 0.002), the major transcription factor regulating the cholesterol homoeostasis, LDL-receptor (*Ldlr*) (CONV+SCD vs. CONV+HFD+Ator: 100.0 ± 11.8% vs. 136.5 ± 13.7%, *p* = 0.078; ABS+SCD vs. ABS+HFD+Ator: 175.9 ± 13.1% vs. 96.3 ± 21.8%, *p* = 0.008) and of proprotein convertase subtilisin kexin typ 9 (*Pcsk9*) (CONV+SCD vs. CONV+HFD+Ator: 100.0 ± 14.4% vs. 101.2 ± 13.9%, *p* = 0.955; ABS+SCD vs. ABS+HFD+Ator: 28.8 ± 3.4% vs. 131.1 ± 53.0%, *p* = 0.03) were differently regulated by atorvastatin in ABS mice as compared to CONV mice. A similar trend was also observed on protein level (Figure 6B) (SREBP2: CONV+HFD vs. CONV+HFD+Ator: 169.8 ± 39.0% (relative protein expression) vs. 213.7 ± 15.5%, *p* = 0.39; ABS+HFD vs. ABS+HFD+Ator: 136.8 ± 31.5% vs. 124.3 ± 33.3%, *p* = 0.81; LDL-R: CONV+HFD vs. CONV+HFD+Ator: 121.4 ± 27.7% vs. 135.7 ± 27.3%, *p* = 0.76; ABS+HFD vs. ABS+HFD+Ator: 178.9± 19.9% vs. 71.2 ± 8.2%, *p* = 0.002). In ileum, mRNA expression of most cholesterol regulating genes displayed a similar trend in both HFD fed CONV and ABS mice with similar alteration upon atorvastatin treatment in both groups (Figure 7). However, mRNA expression of Niemann-Pick C1-like protein 1 (*Npc1l1*), the major transporter protein regulating intestinal cholesterol absorption was significantly increased by atorvastatin in mice with depleted gut microbiota whereas in CONV mice atorvastatin tended to reduce its expression (CONV+HFD vs. CONV+HFD+Ator: 86.5 ± 10.6% vs. 59.0 ± 11.7%, *p* = 0.12; ABS+HFD vs. ABS+HFD+Ator: 80.1 ± 13.5% vs. 119.8 ± 16.8%, *p* = 0.1; CONV+HFD+Ator vs. ABS+HFD+Ator: 59.0 ± 11.7% vs. 119.8 ± 16.8%, *p* = 0.018) suggesting gut microbiota-dependent inverse regulation of intestinal cholesterol absorption by atorvastatin. An overview on the role of the key regulating genes involved in hepatic and intestinal cholesterol metabolism and altered gene expression upon atorvastatin treatment in CONV and ABS mice is provided in Figure 8.

## 4. Discussion

The present study is the first to explore the implication of the gut-microbiome for the lipid modulatory effect of atorvastatin in a gut microbiota depleted mouse model and provides several important observations: (i) Atorvastatin partly reverses the shift in gut microbiota composition induced by high-fat diet, (ii) the lipid lowering effect of atorvastatin is significantly attenuated in mice with depleted gut microbiota, and (iii) depletion of gut microbiota significantly alters the atorvastatin-related effects on distinct hepatic and intestinal genes involved in cholesterol metabolism.

Low response to statin therapy may impede proper control of hypercholesterolemia for optimized cardiovascular disease prevention. However, mechanisms of inter-individual variations, in response to statin therapy, still remain unclear [10]. We hypothesized that the magnitude of statin response may at least be partly related to the gut microbiome, given recent advances in understanding the implication of the gut microbiome and its metabolites for the host’s physiology and metabolism, as well as its interaction with pharmaceutic actions of metabolic drugs.

Accumulating evidence indicates that the gut microbiome and microbiota-dependent metabolites modulate the effect of metabolic drugs [11]. For example, the antidiabetic effects of metformin have been identified to be related to the microbiota. In particular, metformin has been reported to alter gut microbiota composition in humans [12], specifically to reduce the abundance of *Bacteroides fragilis* in the gut, which leads to an increase in antagonists of the farnesoid X receptor (FXR), and ultimately results in improved insulin sensitivity [13]. Another recent study observed significant changes in *Escherichia* and *Intestinibacter* abundance in metformin-treated patients [5], which was also found in another cross-sectional study comparing metformin-treated and untreated patients with type II diabetes [11]. Findings from these studies point to a bi-directional relation between the gut microbiome and drug action: While metformin alters the composition of the gut microbiome, either directly or indirectly, the gut bacterial profile also impacts on the anti-diabetic effects of metformin.

In view of these findings, we aimed to explore whether a cross talk between the gut microbiome and statin therapy may also modulate cholesterol lowering effects of statins. Indeed, we observed reduced cholesterol lowering capacity of atorvastatin in mice with depleted gut microbiome, suggesting the involvement of bacterial pathways in cholesterol regulating actions of atorvastatin. Notably, atorvastatin-related effects on two metabolic genes involved in hepatic cholesterol homeostasis, *Ldlr* and *Srebp2*, were partly different upon gut microbiome depletion, suggesting that gut microbiota-related circuits are involved in classical statin-regulated hepatic cholesterol metabolism. Moreover, the expression of *Npc1l1*, the major transporter protein regulating intestinal cholesterol absorption [14] increased by atorvastatin in mice, with depleted gut microbiome. It tended to decrease upon atorvastatin treatment in conventional mice as compared to abiotic mice, suggesting different cholesterol absorption capacity in the small intestine between conventional and abiotic mice. These differences may as a net effect ultimately lead to attenuated cholesterol regulating property of atorvastatin in abiotic mice as compared to conventional mice.

Importantly, a broader analysis of the lipidomic profile revealed that distinct lipid classes which were decreased by atorvastatin in conventionally raised mice were unaltered upon atorvastatin in gut microbiome depleted mice. In particular, several sphingolipids were no longer different following atorvastatin treatment in abiotic mice. These observations may have additional implication for atheroprotective features of statins given the potential pro-atherogenic effects of sphingolipids and ceramides [15].

Importantly, atorvastatin treated hypercholesterolemic mice demonstrated a shift in the composition of the gut microbiota. While, a high-fat diet decreased the relative abundance of the bacterial phylum *Bacteroidetes* and increased the abundance of *Firmicutes* as compared to standard chow diet, the ratio between *Firmicutes* to *Bacteroidetes* was reversed towards control conditions upon atorvastatin treatment, although not fully restored. This reorganization of the gut microbial community may drive intestinal cholesterol-regulating circuits. Indeed, several strains belonging to *Bacteroidetes* have been identified to possess cholesterol-reducing activity by converting cholesterol to the saturated product coprostanol [16].

A recent study examining the effect of simvastatin on the gut-microbiome and metabolome in mice observed a profound shift in microbiota-related metabolites. In particular, host serum levels of distinct acrylcarnitines were significantly reduced, such as hydroxytetradecenoylcarnitine, octadecanoylcarnitine and malonylcarnitine+3 hydroxybutyrylcarnitine, which are considered to promote obesity and metabolic diseases [17]. Another metabolic pathway that may link the gut microbiome with the magnitude of statin effect are secondary bile acids [18]. In particular, specific primary and secondary bile metabolites that have been correlated with a lipid lowering response to statins, include taurocholic acid, glycholic acid, taurochenodeoxycholic acid, glychoneodeoxycholic acid and glycoursodeoxycholic acid, suggesting a modulatory effect of the gut microbiome on the lipid lowering capability of statins based on their regulation of bile metabolites. 

## 5. Conclusions

Our findings demonstrate that atorvastatin´s modulatory actions on the lipidome including LDL lowering effects partly depend on its interaction with the gut microbiome. This cross-talk may contribute to the inter-individual variation of response to statin therapy depending on the patients’ gut microbial profile. However, additional clinical studies, combining untargeted metabolomics and metaproteomics, are required to identify distinct microbial pathways that impact on statin effects and to determine how they interact with the host targets to regulate cholesterol metabolism.

## Supplementary Materials

The following are available online at https://www.mdpi.com/2077-0383/9/5/1596/s1, Table S1: qPCR primers for 16S rRNA analysis a, Table S2: qPCR primers for hepatic and intestinal analysis, Table S3: Effect of atorvastatin on selected lipids.
