# Supplementary material for: Impact of the Gut Microbiota on Atorvastatin Mediated Effects on Blood Lipids
Source: J Clin Med. 2020 May 25;9(5):1596. doi: 10.3390/jcm9051596 (PMC7290826; doi:10.3390/jcm9051596)
Supplement: Supplementary file 1 [file jcm-09-01596-s001.pdf]

Supplemental Table S1: qPCR primers for 16S rRNA analysis[a]

Target	Reference strain	Primer Sequence (5`to 3`) [b] and Reference
Total Eubacterial load	Escherichia coli ATCC 25922	F: CGGYCCAGACTCCTACGGG<br>R: TTACCGCGGCTGCTGGCAC<br>Ref.: [1]
Clostridium Cluster IV	Clostridium leptum DSMZ 753	F: TTACTGGGTGTAAAGGG<br>R: TAGAGTGCTCTTGCGTA<br>Ref.: [2]
Clostridium coccoides group	Clostridium coccoides DSMZ 935	F: AAATGACGGTACCTGACTAA<br>R: CTTTGAGTTTCATTCTTGCGAA<br>Ref.: [3]
Bacteroides/Prevotella spp.	Bacteroides ovatus DSMZ 1896	F: GAAGGTCCCCCACATTG<br>R: CAATCGGAGTTCTTCGTG<br>Ref.: [4]
γ-Proteobacteria/ Enterobacteriaceae	Escherichia coli ATCC 25922	F: AAACTCAAATGAATTGACGG<br>R: CTTTTGCAACCCACTCC<br>Ref.: [5]
Lactobacillus group	Lactobacillus acidophilus DSM 20079	F: CACCGCTACACATGGAG<br>R: AGCAGTAGGGAATCTTCCA<br>Ref.: [6,7]
Bifidobacterium genus	Bifidobacterium sp. (murine origin)	F: CTCCTGGAAACGGGTGG<br>R: GGTGTTCTTCCCGATATCTACA<br>Ref.: [3,6,7]
Enterococcus genus	Enterococcus faecalis DSM 20478	F: CCTTATTGTTAGTTGCCATCATT<br>R: ACTCGTTGTACTTCCCATTGT<br>Ref.: [8]
Mouse Intestinal Bacteroides	MIB plasmid 16-1	F: CCAGCAGCCGCGGTAATA<br>R: CGCATTCCGCATACTTCTC<br>Ref.: [9]

Supplemental Table 1: [a] – modified from: Ref. [10]. [b] – F = Forward, R = Reverse.

Supplemental Table S2: qPCR primers for hepatic and intestinal analysis

Gene	Species	Assay-ID	RefSeq
Abca1	Mouse	Mm00442646_m1	NM_013454.3
Abcg5	Mouse	Mm00446241_m1	NM_031884.1
Acat2	Mouse	Mm00782408_s1	NM_009338.3
Asbt	Mouse	Mm00488258_m1	NM_011388.2
Cyp7a1	Mouse	Mm00484150_m1	NM_007824.2
Fxr	Mouse	Mm00436425_m1	NM_001163504.1<br>NM_001163700.1<br>NM_009108.2
Gapdh	Mouse	Mm99999915_g1	NM_001289726.1<br>NM_008084.3
Hmgcr	Mouse	Mm01282499_m1	NM_008255.2
Ldlr	Mouse	Mm01177349_m1	NM_001252658.1<br>NM_001252659.1<br>NM_010700.3
Npc1l1	Mouse	Mm01191973_m1	NM_207242.2
Pcsk9	Mouse	Mm01263610_m1	NM_153565.2
Srb1	Mouse	Mm00450234_m1	NM_001205082.1<br>NM_001205083.1<br>NM_016741.2
Srebp2	Mouse	Mm01306292_m1	NM_033218.1

Supplemental Table S3: Effect of atorvastatin on selected lipids

CONV – Glycerophospholipids

Metabolite	SCD	HFD	HFD + Ator	p: SCD : HFD	p: SCD : HFD + Ator	p: HFD : HFD + Ator
**lysoPC a**						
**C16:0**	187,8 ± 8,0	154,4 ± 10,9	126,4 ± 11,7	0,052	0,005	0,119
**C16:1**	9,8 ± 0,9	6,4 ± 0,4	5,6 ± 0,5	0,009	0,004	0,287
**C17:0**	2,3 ± 0,1	7,8 ± 0,5	6,7 ±0,5	<0,001	<0,001	0,157
**C18:0**	79,0 ± 5,4	115,9 ± 9,8	104,0 ± 9,1	0,018	0,064	0,4
**C18:1**	77,5 ± 4,5	109,6 ± 7,5	89,6 ± 7,7	0,012	0,248	0,101
**C18:2**	111,0 ± 5,2	135,9 ± 14,2	99,4 ± 6,2	0,18	0,207	0,046
**C20:3**	8,9 ± 0,7	12,6 ± 1,0	10,6 ± 1,1	0,027	0,255	0,216
**C20:4**	42,3 ± 5,0	51,6 ± 4,1	48,6 ± 4,7	0,193	0,391	0,643
**C24:0**	0,5 ± 0,0	0,7 ± 0,2	0,5 ± 0,1	0,37	0,755	0,32
**C28:0**	0,3 ± 0,0	0,5 ± 0,1	0,5 ± 0,1	0,058	0,148	0,658
**PC aa**						
**C28:1**	0,3 ± 0,0	0,6 ± 0,1	0,3± 0,0	0,071	0,027	0,119
**C30:0**	0,6 ± 0,0	0,9 ± 0,1	0,7 ± 0,0	0,047	0,064	0,14
**C32:0**	9,5 ± 0,8	7,1 ± 0,6	6,1 ± 0,4	0,049	0,005	0,248
**C32:1**	6,2 ± 1,0	4,1 ± 0,4	3,9 ± 0,3	0,07	0,04	0,579
**C32:2**	0,9 ± 0,1	0,7 ± 0,1	0,6 ± 0,1	0,134	0,015	0,16
**C32:3**	0,1 ± 0,0	0,2 ± 0,0	0,2 ± 0,0	0,006	0,128	0,148
**C34:1**	97,4 ± 9,9	126,2 ± 8,9	112,7 ± 7,6	0,067	0,251	0,278
**C34:2**	184,5 ± 7,5	187,4 ± 19,1	153,6 ± 6,9	0,9	0,019	0,134
**C34:3**	8,4 ± 0,4	8,2 ± 0,9	6,7 ± 0,5	0,817	0,031	0,159
**C34:4**	0,5 ± 0,0	0,5 ± 0,1	0,5 ± 0,0	0,786	0,835	0,646
**C36:0**	0,9 ± 0,1	1,7 ± 0,1	1,5 ± 0,2	0,002	0,037	0,359
**C36:1**	22,0 ± 3,5	74,9 ± 5,7	60,8 ± 4,7	<0,001	<0,001	0,091
**C36:2**	133,0 ± 6,6	196,6 ± 14,6	170,2 ± 5,0	0,009	0,003	0,127
**C36:3**	58,4 ± 0,3	70,4 ± 7,0	60,3 ± 2,8	0,247	0,627	0,219
**C36:4**	93,3 ± 9,3	92,4 ± 9,4	87,3 ± 8,8	0,951	0,654	0,7
**C36:5**	5,3 ± 0,3	5,2 ± 0,5	5,1 ± 0,6	0,873	0,786	0,899
**C36:6**	0,4 ± 0,0	0,4 ± 0,0	0,3 ± 0,0	0,869	0,295	0,47
**C38:0**	1,0 ± 0,1	1,7 ± 0,1	1,4 ± 0,1	<0,001	0,036	0,19
**C38:3**	14,6 ± 1,0	24,8 ± 2,2	23,4 ± 2,4	0,006	0,018	0,671
**C38:4**	61,5 ± 6,5	91,7 ± 7,9	96,3 ± 7,5	0,025	0,011	0,685
**C38:5**	25,9 ± 2,4	28,4 ± 1,7	30,9 ± 2,4	0,41	0,188	0,423
**C38:6**	69,3 ± 7,0	69,1 ± 5,3	66,6 ± 6,0	0,986	0,779	0,76
**C40:2**	0,1 ± 0,0	0,2 ± 0,0	0,1 ± 0,0	0,086	0,998	0,039
**C40:3**	0,1 ± 0,0	0,2 ± 0,0	0,2 ± 0,0	0,02	0,009	0,455
**C40:4**	0,9 ± 0,0	1,3 ± 0,1	1,3 ± 0,1	0,002	0,018	0,749
**C40:5**	2,2 ± 0,1	2,2 ± 0,0	2,4 ± 0,2	0,284	0,285	0,507
**C40:6**	25,7 ± 1,4	37,4 ± 2,6	37,0 ± 3,3	0,008	0,023	0,93
**C42:1**	0,1 ± 0,0	0,1 ± 0,0	0,1 ± 0,0	0,123	0,211	0,233
**C42:4**	0,1 ± 0,0	0,1 ± 0,0	0,1 ± 0,0	0,306	0,249	0,992
**C42:5**	0,1 ± 0,0	0,1 ± 0,0	0,1 ± 0,0	0,135	0,313	0,566
**PC ae**						
**C30:2**	0,1 ± 0,0	0,1 ± 0,0	0,1 ± 0,0	0,142	<0,001	0,299
**C32:1**	0,3 ± 0,0	0,5 ± 0,1	0,4 ± 0,0	0,142	0,082	0,338

**C32:2**	0,1 ± 0,0	0,2 ± 0,0	0,2 ± 0,0	0,031	0,038	0,184
**C34:0**	0,3 ± 0,0	1,2 ± 0,1	0,9 ± 0,0	0,001	<0,001	0,079
**C34:1**	2,5 ± 0,2	4,9 ± 0,4	4,4 ± 0,3	0,001	0,001	0,332
**C34:2**	1,4 ± 0,1	2,0 ± 0,2	1,7 ± 0,1	0,02	0,085	0,116
**C34:3**	0,4 ± 0,0	0,5 ± 0,0	0,4 ± 0,0	0,015	0,992	0,042
**C36:0**	0,2 ± 0,0	0,6 ± 0,0	0,4 ± 0,1	<0,001	0,021	0,04
**C36:1**	1,6 ± 0,2	7,8 ± 0,6	6,9 ± 0,4	<0,001	<0,001	0,251
**C36:2**	4,1 ± 0,3	13,1 ± 1,3	10,6 ± 0,4	<0,001	<0,001	0,096
**C36:3**	1,0 ± 0,1	2,2 ± 0,2	1,8 ± 0,1	0,002	<0,001	0,173
**C36:4**	2,2 ± 0,4	2,4 ± 0,2	2,1 ± 0,2	0,667	0,911	0,41
**C36:5**	1,1 ± 0,1	1,0 ± 0,1	0,9 ± 0,1	0,778	0,425	0,435
**C38:0**	2,0 ± 0,2	2,2 ± 0,1	2,2 ± 0,2	0,436	0,527	0,968
**C38:1**	0,5 ± 0,1	1,3 ± 0,1	1,2 ± 0,1	<0,001	<0,001	0,3
**C38:2**	1,5 ± 0,0	2,3 ± 0,2	1,9 ± 0,1	0,005	0,027	0,132
**C38:3**	1,0 ± 0,1	2,3 ± 0,2	2,1 ± 0,2	<0,001	<0,001	0,517
**C38:4**	2,4 ± 0,2	6,4 ± 0,4	5,9 ± 0,1	<0,001	<0,001	0,375
**C38:5**	2,4 ± 0,3	3,1 ± 0,2	3,0 ± 0,3	0,096	0,164	0,797
**C38:6**	1,1 ± 0,1	1,4 ± 0,1	1,2 ± 0,1	0,112	0,618	0,205
**C40:1**	2,3 ± 0,1	2,5 ± 0,2	2,3 ± 0,2	0,359	0,813	0,574
**C40:2**	0,3 ± 0,0	0,6 ± 0,0	0,5 ± 0,1	0,004	0,009	0,979
**C40:3**	0,3 ± 0,0	0,6 ± 0,0	0,6 ± 0,1	0,002	0,004	0,49
**C40:4**	0,7 ± 0,1	1,1 ± 0,1	1,1 ± 0,1	0,017	0,012	0,728
**C40:5**	0,6 ± 0,0	1,0 ± 0,1	1,0 ± 0,1	0,003	0,005	0,918
**C40:6**	1,4 ± 0,1	4,4 ± 0,2	4,2 ± 0,3	<0,001	<0,001	0,618
**C42:1**	0,3 ± 0,0	0,3 ± 0,0	0,3 ± 0,0	0,9	0,446	0,299
**C42:2**	0,4 ± 0,0	0,5 ± 0,0	0,4 ± 0,0	0,004	0,186	0,2
**C42:3**	1,1 ± 0,1	1,1 ± 0,1	1,1 ± 0,1	0,91	0,912	0,973
**C44:3**	0,1 ± 0,0	0,2 ± 0,0	0,1 ± 0,0	0,014	0,167	0,069
**C44:5**	0,2 ± 0,0	0,2 ± 0,0	0,2 ± 0,0	0,966	0,331	0,539

ABS – Glycerophospholipids

Metabolite	SCD	HFD	HFD + Ator	p: SCD : HFD	p: SCD : HFD + Ator	p: HFD : HFD + Ator
**lysoPC a**						
**C16:0**	212,3 ± 32,6	130,3 ± 3,9	112,4 ± 4,3	0,047	0,011	0,02
**C16:1**	7,4 ± 1,0	5,4 ± 0,3	5,0 ± 0,4	0,11	0,043	0,412
**C17:0**	3,1 ± 0,3	6,5 ± 0,2	5,8 ± 0,2	<0,001	<0,001	0,1
**C18:0**	101,3 ± 15,9	101,3 ± 3,4	93,9 ± 2,6	0,998	0,622	0,119
**C18:1**	66,2 ± 12,9	88,6 ± 8,4	85,8 ± 4,9	0,198	0,164	0,775
**C18:2**	133 ± 12,9	114,7 ± 15,4	102,6 ± 10,9	0,398	0,114	0,513
**C20:3**	8,3 ± 1,5	10,4 ± 1,5	10,3 ± 0,7	0,366	0,229	0,953
**C20:4**	49,5 ± 10,2	33,1 ± 1,4 b	31,6 ± 1,4 b	0,162	0,09	0,49
**C24:0**	0,6 ± 0,0	0,5 ± 0,0	0,5 ± 0,0	0,092	0,381	0,31
**C28:0**	0,5 ± 0,1	0,4 ± 0,1	0,5 ± 0,1	0,607	0,941	0,423
**PC aa**						
**C28:1**	0,4 ± 0,1	0,4 ± 0,0	0,4 ± 0,0 b	0,928	0,743	0,383
**C30:0**	0,9 ± 0,1 a	0,7 ± 0,1	0,7 ± 0,0	0,184	0,075	0,91
**C32:0**	10,6 ± 1,0	6,2 ± 0,0	5,6 ± 0,2	0,01	<0,001	0,071
**C32:1**	4,4 ± 0,5	3,7 ± 0,0	5,4 ± 0,9	0,312	0,4	0,211
**C32:2**	0,8 ± 0,1	0,7 ± 0,1	0,8 ± 0,1	0,45	0,685	0,69
**C32:3**	0,1 ± 0,0	0,2 ± 0,01	0,2 ± 0,0 a	0,015	0,002	0,609
**C34:1**	89,3 ± 11,9	119,7 ± 0,9	142,8 ± 9,5 a	0,084	0,009	0,119

**C34:2**	220,8±10,8 a	184 ± 15,4	187,4 ± 9,9 a	0,099	0,057	0,852
**C34:3**	8,9 ± 0,8	7,8 ± 0,9	8,9 ± 1,1	0,514	0,835	0,481
**C34:4**	0,4 ± 0,1	0,5 ± 0,0	0,5 ± 0,1	0,457	0,203	0,518
**C36:0**	1,2 ± 0,1	1,9 ± 0,2	1,4 ± 0,1	0,015	0,284	0,02
**C36:1**	21 ± 2,5	70,9 ± 11	76,7 ± 3,4 a	0,005	< 0,001	0,519
**C36:2**	164 ± 3,8 b	205 ± 18,5	204,2 ± 8,5 b	0,073	0,006	0,968
**C36:3**	58,9 ±7,3	65,1 ± 7,9	70,3 ± 5,7	0,585	0,249	0,6
**C36:4**	100,2 ± 11,4	72,2 ± 3,9	77,9 ± 5,5	0,06	0,1	0,447
**C36:5**	5,1 ± 0,4	5,8 ± 0,6	6,9 ± 0,6	0,366	0,061	0,265
**C36:6**	0,3 ± 0,0	0,3 ± 0,0	0,4 ± 0,0	0,457	0,088	0,231
**C38:0**	1,0 ± 0,0	1,4 + 0,0 a	1,3 ± 0,1	0,004	0,006	0,821
**C38:3**	15,1 ± 1,5	23,5 ± 3,2	25,9 ± 1,4	0,053	0,001	0,482
**C38:4**	68,1 ± 7,1	70,9 ± 3,8	77,2 ± 3,9	0,739	0,273	0,294
**C38:5**	23,9 ± 3,7	22,0 ± 1,9 a	25,8 ± 1,5	0,678	0,618	0,164
**C38:6**	72,5 ± 8,0	61,3 ± 4,2	61,5 ± 5,3	0,264	0,273	0,976
**C40:2**	0,2 ± 0,0	0,1 ± 0,0	0,2 ± 0,0	0,11	0,4	0,374
**C40:3**	0,2 ± 0,0 a	0,2 ± 0,0	0,2 ± 0,0	0,212	0,844	0,211
**C40:4**	1,1 ± 0,1	1,1 ± 0,1	1,2 ± 0,0	0,976	0,523	0,276
**C40:5**	2,5 ± 0,6	2,0 ± 0,1 a	2,4 ± 0,2	0,393	0,798	0,143
**C40:6**	27,7 ± 2,1	32,3 ± 2,4	35,7 ± 2,7	0,197	0,062	0,395
**C42:1**	0,1 ± 0,0	0,1 ± 0,0	0,1 ± 0,0	0,795	0,886	0,659
**C42:4**	0,1 ± 0,0	0,1 ± 0,0	0,1 ± 0,0 a	0,039	0,016	0,678
**C42:5**	0,1 ± 0,0 c	0,1 ± 0,0 a	0,1 ± 0,0	< 0,001	0,002	0,178
**PC ae**						
**C30:2**	0,1 ± 0,0 a	0,1 ± 0,0	0,1 ± 0,0	0,26	0,181	0,731
**C32:1**	0,4 ± 0,0 a	0,4 ± 0,0	0,4 ± 0,0	0,727	0,619	0,838
**C32:2**	0,3 ±0,1 a	0,2 ± 0,0	0,2 ± 0,0	0,784	0,236	0,001
**C34:0**	0,4 ± 0,0	0,9 ± 0,0	0,9 ± 0,1	<0,001	<0,001	0,976
**C34:1**	2,6 ± 0,1	4,4 ± 0,1	5,1 ± 0,2	<0,001	<0,001	0,018
**C34:2**	1,6 ± 0,1	2,2 ± 0,2	2,2 ± 0,2 a	0,058	0,027	0,927
**C34:3**	0,6 ± 0,0 b	0,6 ± 0,0	0,7 ± 0,0 c	0,553	0,046	0,08
**C36:0**	0,3 ± 0,0	0,5 ± 0,0 a	0,5 ± 0,0	0,005	0,028	0,921
**C36:1**	1,9 ± 0,2	8,0 ± 1,2	8,8 ± 0,5 a	0,002	<0,001	0,539
**C36:2**	5,6 ± 0,3 a	12,4 ± 1,8	13,9 ± 1,0 a	0,009	<0,001	0,46
**C36:3**	1,0 ± 0,1	1,9 ± 0,3	2,1 ± 0,2	0,039	0,002	0,561
**C36:4**	1,7 ± 0,2	2,0 ± 0,1	2,1 ± 0,1	0,182	0,492	0,506
**C36:5**	1,4 ± 0,1	1,2 ± 0,1	1,2 ± 0,0	0,298	0,168	0,936
**C38:0**	1,8 ± 0,2	1,8 ± 0,1	2,0 ± 0,1	0,915	0,567	0,338
**C38:1**	0,7 ± 0,0 a	1,1 ± 0,2	1,3 ± 0,1	0,036	<0,001	0,432
**C38:2**	1,9 ± 0,0 c	2,1 ± 0,2	2,2 ± 0,2	0,628	0,295	0,678
**C38:3**	1,1 ± 0,1	2,2 ± 0,3	2,4 ± 0,1	0,017	<0,001	0,471
**C38:4**	2,6 ± 0,3	5,0 ± 0,3 a	5,4 ± 0,2	0,001	<0,001	0,251
**C38:5**	2,1 ± 0,2	2,5 ± 0,0	2,7 ± 0,1	0,274	0,061	0,248
**C38:6**	1,1 ± 0,1	1,3 ± 0,1	1,4 ± 0,1	0,121	0,08	0,573
**C40:1**	1,6 ± 0,2 a	1,5 ± 0,2 b	1,6 ± 0,1 a	0,643	0,962	0,536
**C40:2**	0,4 ± 0,0	0,5 ± 0,0	0,5 ± 0,0	0,026	0,013	0,494
**C40:3**	0,4 ± 0,0	0,5 ± 0,0	0,5 ± 0,0	0,133	0,039	0,443
**C40:4**	0,8 ± 0,1	0,8 ± 0,1	0,9 ± 0,0	0,691	0,268	0,575
**C40:5**	0,6 ± 0,1	0,7 ± 0,0 a	0,8 ± 0,1 a	0,25	0,166	0,566
**C40:6**	1,4 ± 0,1	3,7 ± 0,2 a	3,9 ± 0,3	<0,001	<0,001	0,555
**C42:1**	0,3 ± 0,0	0,2 ± 0,0 a	0,2 ± 0,0	0,039	0,073	0,034
**C42:2**	0,3 ± 0,0	0,3 ± 0,0 a	0,4 ± 0,0	0,5	<0,001	0,058
**C42:3**	0,7 ± 0,0 b	0,7 ± 0,1 b	0,7 ± 0,0 a	0,7	0,36	0,261
**C44:3**	0,1 ± 0,0 a	0,1 ± 0,0	0,1 ± 0,0	0,638	0,372	0,254

**C44:5**	0,2 ± 0,0 b	0,2 ± 0,0	0,2 ± 0,0	0,897	0,133	0,4

CONV – Triacylglycerols

Metabolite	SCD	HFD	HFD + Ator	p: SCD : HFD	p: SCD : HFD + Ator	p: HFD : HFD + Ator
**TG (14:0)**						
**34:1**	2,6 ± 0,4	3,3 ± 0,7	2,4 ± 0,5	0,577	0,78	0,388
**34:2**	1,4 ± 0,4	1,4 ± 0,4	0,8 ± 0,1	0,929	0,153	0,171
**36:1**	1,1 ± 0,1	3,3 ± 0,6	2,4 ± 0,3	0,012	0,007	0,166
**36:2**	2,7 ± 0,4	3,3 ± 0,9	2,4 ± 0,5	0,58	0,619	0,375
**36:3**	1,4 ± 0,3	1,2 ± 0,4	0,8 ± 0,2	0,646	0,09	0,37
**36:4**	0,4 ± 0,0	0,4 ± 0,0	0,2 ± 0,0	0,273	0,07	0,048
**TG (16:0)**						
**32:1**	9,4 ± 1,7	6,7 ± 1,6	4,8 ± 1,1	0,362	0,089	0,403
**32:2**	3,1 ± 1,0	2,0 ± 0,4	1,5 ± 0,3	0,346	0,222	0,355
**32:3**	0,3 ± 0,1	0,4 ± 0,1	0,2 ± 0,0	0,734	0,121	0,238
**34:1**	27,2 ± 9,7	32,6 ± 8,3	18,3 ± 2,8	0,687	0,309	0,113
**34:2**	29,2 ± 8,5	15,2 ± 4,1	11,6 ± 1,6	0,157	0,056	0,429
**34:3**	6,2 ± 1,3	2,5 ± 0,7	1,9 ± 0,3	0,032	0,009	0,451
**34:4**	0,5 ± 0,1	0,3 ± 0,1	0,3 ± 0,0	0,141	0,015	0,344
**35:2**	1,1 ± 0,3	2,4 ± 0,6	1,8 ± 0,3	0,098	0,121	0,351
**35:3**	0,3 ± 0,1	0,5 ± 0,1	0,4 ± 0,1	0,213	0,457	0,383
**36:2**	55,1 ± 10,2	60,3 ± 14,9	44,3 ± 6,7	0,794	0,387	0,355
**36:3**	30,2 ± 6,1	18,8 ± 4,7	14,9 ± 2,0	0,178	0,034	0,465
**36:4**	10,5 ± 3,1	3,2 ± 0,9	2,2 ± 0,3	0,042	0,019	0,265
**36:5**	2,1 ± 0,8	0,5 ± 0,1	0,3 ± 0,0	0,051	0,031	0,202
**37:3**	0,3 ± 0,1	0,5 ± 0,1	0,2 ± 0,0	0,204	0,076	0,026
**38:2**	1,2 ± 0,3	1,3 ± 0,2	1,1 ± 0,2	0,808	0,724	0,426
**38:3**	1,3 ± 0,3	1,1 ± 0,3	0,9 ± 0,1	0,498	0,115	0,531
**38:4**	1,4 ± 0,3	1,4 ± 0,3	1,1 ± 0,1	0,949	0,249	0,31
**38:5**	1,6 ± 0,4	1,1 ± 0,2	0,9 ± 0,1	0,241	0,062	0,42
**38:6**	0,9 ± 0,2	0,7 ± 0,2	0,5 ± 0,0	0,494	0,116	0,455
**40:6**	1,0 ± 0,2	0,8 ± 0,2	0,6 ± 0,1	0,389	0,016	0,499
**40:7**	1,3 ± 0,3	1,7 ± 0,4	1,2 ± 0,1	0,435	0,824	0,196
**40:8**	0,5 ± 0,2	0,5 ± 0,1	0,3 ± 0,0	0,91	0,313	0,167
**TG (16:1)**						
**34:0**	2,1 ± 0,7	2,2 ± 0,5	1,3 ± 0,2	0,911	0,236	0,118
**34:1**	20,4 ± 5,8	10,2 ± 2,6	7,9 ± 1,2	0,125	0,05	0,4
**34:2**	10,8 ± 1,6	5,1 ± 0,8	3,7 ± 0,6	0,02	0,003	0,205
**34:3**	1,7 ± 0,1	0,6 ± 0,1	0,5 ± 0,1	0,001	<0,001	0,6
**36:1**	2,2 ± 0,2	3,0 ± 0,7	2,3 ± 0,4	0,376	0,923	0,379
**36:2**	13,9 ± 1,2	12,5 ± 2,6	11,0 ± 2,0	0,672	0,294	0,677
**36:3**	5,9 ± 1,0	3,3 ± 0,7	2,9 ± 0,5	0,065	0,024	0,7
**36:4**	1,6 ± 0,3	0,5 ± 0,1	0,4 ± 0,1	0,007	0,003	0,645
**38:3**	0,4 ± 0,1	0,3 ± 0,0	0,3 ± 0,0	0,24	0,359	0,624

**38:4**	0,5 ± 0,0	0,4 ± 0,1	0,3 ± 0,0	0,186	0,006	0,624
**38:5**	0,5 ± 0,1	0,3 ± 0,1	0,3 ± 0,0	0,192	0,057	0,442
**TG (17:0)**						
**34:2**	0,5 ± 0,0	0,9 ± 0,2	0,7 ± 0,1	0,185	0,13	0,431
**36:3**	0,4 ± 0,0	0,9 ± 0,2	0,7 ± 0,1	0,1	0,039	0,57
**36:4**	0,3 ± 0,1	0,2 ± 0,0	0,2 ± 0,0	0,768	0,14	0,085
**TG (17:1)**						
**34:1**	1,0 ± 0,2	2,1 ± 0,5	1,4 ± 0,2	0,12	0,161	0,261
**34:2**	0,6 ± 0,1	0,9 ± 0,1	0,7 ± 0,0	0,021	0,143	0,074
**36:3**	0,3 ± 0,0	0,5 ± 0,1	0,4 ± 0,1	0,263	0,282	0,625
**TG (18:0)**						
**32:2**	0,2 ± 0,0	0,7 ± 0,2	0,5 ± 0,1	0,069	0,093	0,442
**34:2**	2,1 ± 0,5	4,3 ± 1,2	2,9 ± 0,4	0,181	0,25	0,329
**34:3**	0,5 ± 0,1	0,7 ± 0,2	0,6 ± 0,1	0,41	0,598	0,526
**36:3**	3,2 ± 0,8	4,5 ± 1,3	3,3 ± 0,6	0,449	0,961	0,406
**36:4**	2,2 ± 0,8	1,0 ± 0,3	0,7 ± 0,1	0,15	0,057	0,332
**38:6**	0,2 ± 0,0	0,3 ± 0,0	0,2 ± 0,0	0,075	0,246	0,132
**TG (18:1)**						
**30:0**	2,8 ± 0,2	4,3 ± 0,9	3,3 ± 0,7	0,315	0,637	0,429
**30:1**	1,7 ± 0,2	1,9 ± 0,2	1,4 ± 0,2	0,559	0,467	0,218
**32:0**	10,3 ± 3,2	15,4 ± 4,7	10,1 ± 1,8	0,425	0,972	0,329
**32:1**	22,7 ± 5,5	15,8 ± 3,9	10,9 ± 1,9	0,334	0,062	0,294
**32:2**	4,5 ± 0,5	3,6 ± 0,6	2,5 ± 0,5	0,276	0,021	0,16
**32:3**	0,2 ± 0,0	0,4 ± 0,1	0,3 ± 0,1	0,262	0,493	0,56
**33:2**	0,6 ± 0,1	1,1 ± 0,3	0,7 ± 0,1	0,164	0,466	0,227
**34:1**	92,6 ± 17,3	123,6 ± 20,4	93,3 ± 1,3	0,29	0,974	0,266
**34:2**	47,0 ± 5,9	35,5 ± 8,4	29,4 ± 4,2	0,327	0,041	0,531
**34:3**	7,8 ± 1,4	4,9 ± 1,2	3,8 ± 0,7	0,159	0,032	0,471
**34:4**	0,7 ± 0,1	0,5 ± 0,1	0,3 ± 0,1	0,201	0,005	0,387
**35:2**	1,2 ± 0,0	3,7 ± 0,8	3,0 ± 0,5	0,068	0,035	0,469
**35:3**	0,4 ± 0,0	0,7 ± 0,2	0,5 ± 0,1	0,329	0,469	0,414
**36:3**	34,1 ± 6,8	22,9 ± 4,6	18,5 ± 2,4	0,221	0,073	0,431
**36:4**	15,1 ± 5,0	4,6 ± 1,0	3,0 ± 0,4	0,087	0,029	0,141
**36:5**	3,1 ± 1,1	0,7 ± 0,2	0,5 ± 0,1	0,038	0,028	0,414
**38:5**	1,9 ± 0,3	2,0 ± 0,5	1,6 ± 0,2	0,869	0,46	0,476
**38:6**	1,6 ± 0,4	1,6 ± 0,4	1,4 ± 0,1	0,966	0,545	0,602
**38:7**	0,4 ± 0,1	0,5 ± 0,1	0,3 ± 0,0	0,528	0,445	0,269
**TG (18:2)**						
**30:0**	0,9 ± 0,3	0,8 ± 0,2	0,7 ± 0,1	0,819	0,591	0,711
**30:1**	0,5 ± 0,1	0,5 ± 0,1	0,4 ± 0,1	0,67	0,414	0,597
**32:0**	4,4 ± 1,5	3,4 ± 1,0	2,5 ± 0,4	0,567	0,185	0,409
**32:1**	5,4 ± 1,2	3,0 ± 0,7	2,1 ± 0,4	0,133	0,024	0,265
**32:2**	1,2 ± 0,2	0,7 ± 0,2	0,4 ± 0,0	0,093	0,006	0,117
**33:0**	0,3 ± 0,0	0,6 ± 0,2	0,4 ± 0,1	0,148	0,167	0,465
**33:1**	0,6 ± 0,1	1,0 ± 0,2	0,6 ± 0,1	0,3	0,683	0,246
**34:0**	3,4 ± 0,9	3,7 ± 1,1	2,7 ± 0,4	0,864	0,446	0,421
**34:1**	26,7 ± 5,7	17,7 ± 4,7	13,7 ± 2,0	0,256	0,05	0,458
**34:2**	18,5 ± 4,7	5,3 ± 1,3	4,1 ± 0,6	0,02	0,011	0,448
**34:3**	3,1 ± 0,8	0,7 ± 0,2	0,5 ± 0,1	0,016	0,023	0,466
**35:1**	0,5 ± 0,1	1,0 ± 0,3	0,7 ± 0,1	0,217	0,31	0,378
**35:2**	0,5 ± 0,1	0,6 ± 0,1	0,4 ± 0,1	0,689	0,489	0,381

**36:0**	0,5 ± 0,1	0,9 ± 0,3	0,6 ± 0,1	0,191	0,345	0,333
**36:1**	4,7 ± 1,1	4,9 ± 1,2	3,5 ± 0,6	0,913	0,346	0,333
**36:2**	18,2 ± 3,7	12,5 ± 2,6	8,4 ± 1,3	0,257	0,028	0,175
**36:3**	20,8 ± 7,7	3,7 ± 0,8	2,6 ± 0,2	0,119	0,102	0,275
**38:4**	0,7 ± 0,2	0,4 ± 0,1	0,3 ± 0,0	0,078	0,043	0,573
**38:5**	0,7 ± 0,2	0,4 ± 0,1	0,3 ± 0,0	0,139	0,081	0,102
**38:6**	0,8 ± 0,3	0,4 ± 0,1	0,3 ± 0,0	0,175	0,023	0,347
**TG (18:3)**						
**32:0**	0,7 ± 0,1	0,6 ± 0,1	0,3 ± 0,0	0,635	0,014	0,088
**32:1**	0,5 ± 0,1	0,4 ± 0,1	0,3 ± 0,0	0,823	0,09	0,127
**34:0**	0,5 ± 0,1	0,4 ± 0,1	0,3 ± 0,1	0,835	0,232	0,477
**34:1**	3,0 ± 0,7	1,9 ± 0,5	1,3 ± 0,2	0,218	0,032	0,312
**34:2**	2,3 ± 0,7	0,7 ± 0,2	0,5 ± 0,1	0,031	0,014	0,323
**36:1**	0,6 ± 0,1	0,6 ± 0,2	0,4 ± 0,1	0,888	0,21	0,212
**36:2**	2,6 ± 0,5	1,4 ± 0,4	1,0 ± 0,1	0,09	0,009	0,281
**36:3**	3,1 ± 1,2	0,4 ± 0,1	0,3 ± 0,0	0,04	0,06	0,367
**TG (20:1)**						
**34:1**	1,1 ± 0,2	1,2 ± 0,3	1,1 ± 0,2	0,706	0,993	0,691
**34:2**	0,9 ± 0,2	0,5 ± 0,1	0,5 ± 0,1	0,115	0,069	0,944
**TG (20:2)**						
**34:1**	1,0 ± 0,2	0,9 ± 0,2	0,7 ± 0,1	0,93	0,339	0,373
**TG (20:3)**						
**32:1**	0,4 ± 0,1	0,3 ± 0,1	0,2 ± 0,0	0,266	0,04	0,266
**34:0**	0,3 ± 0,1	0,4 ± 0,1	0,3 ± 0,0	0,564	0,596	0,298
**34:1**	1,8 ± 0,4	2,8 ± 0,6	1,9 ± 0,3	0,246	0,884	0,23
**34:2**	1,1 ± 0,1	1,0 ± 0,2	0,9 ± 0,1	0,934	0,227	0,627
**36:3**	0,8 ± 0,2	0,7 ± 0,1	0,5 ± 0,1	0,587	0,214	0,45
**36:4**	0,3 ± 0,1	0,2 ± 0,1	0,2 ± 0,0	0,66	0,209	0,359
**TG (20:4)**						
**32:0**	0,5 ± 0,2	0,4 ± 0,1	0,4 ± 0,1	0,706	0,5	0,684
**32:1**	0,8 ± 0,3	0,4 ± 0,1	0,3 ± 0,0	0,215	0,086	0,31
**34:0**	0,6 ± 0,2	0,7 ± 0,2	0,5 ± 0,0	0,688	0,608	0,247
**34:1**	4,2 ± 1,3	3,0 ± 0,7	2,2 ± 0,3	0,432	0,143	0,34
**34:2**	2,6 ± 0,9	1,2 ± 0,3	1,0 ± 0,1	0,127	0,07	0,448
**34:3**	0,5 ± 0,1	0,2 ± 0,0	0,2 ± 0,0	0,052	0,015	0,227
**36:2**	2,7 ± 0,5	2,4 ± 0,5	2,1 ± 0,2	0,656	0,244	0,583
**36:3**	1,7 ± 0,5	0,9 ± 0,2	0,7 ± 0,1	0,125	0,049	0,293
**36:4**	1,0 ± 0,4	0,3 ± 0,1	0,2 ± 0,1	0,0687	0,197	0,535
**TG (20:5)**						
**34:1**	0,6 ± 0,1	0,6 ± 0,1	0,5 ± 0,1	0,763	0,433	0,461
**34:2**	0,4 ± 0,1	0,3 ± 0,0	0,3 ± 0,1	0,353	0,171	0,322
**36:2**	0,4 ± 0,0	0,6 ± 0,1	0,5 ± 0,1	0,181	0,53	0,32
**36:3**	0,3 ± 0,0	0,2 ± 0,1	0,2 ± 0,1	0,241	0,133	0,763
**TG (22:5)**						
**34:1**	1,5 ± 0,4	1,2 ± 0,3	1,0 ± 0,1	0,578	0,221	0,539
**34:2**	0,8 ± 0,2	0,5 ± 0,1	0,4 ± 0,1	0,149	0,077	0,844
**TG (22:6)**						
**32:0**	0,6 ± 0,2	0,8 ± 0,2	0,7 ± 0,1	0,585	0,611	0,639

**32:1**	0,9 ± 0,2	0,8 ± 0,2	0,6 ± 0,1	0,849	0,311	0,346
**34:1**	4,3 ± 1,2	6,1 ± 1,5	5,2 ± 0,5	0,381	0,461	0,573
**34:2**	2,4 ± 0,8	1,9 ± 0,4	1,8 ± 0,1	0,599	0,413	0,761
**34:3**	0,4 ± 0,1	0,3 ± 0,1	0,2 ± 0,0	0,385	0,3	0,684

ABS – Triacylglycerols

Metabolite	SCD	HFD	HFD + Ator	p: SCD : HFD	p: SCD : HFD + Ator	p: HFD : HFD + Ator
**TG (14:0)**						
**34:1**	1,6 ± 0,1	3,3 ± 1,3	2,0 ± 0,3	0,402	0,452	0,257
**34:2**	1,4 ± 0,2	1,2 ± 0,4	0,8 ± 0,1	0,636	0,036	0,35
**36:1**	0,9 ± 0,1	3,2 ± 1,0	2,7 ± 0,2	0,064	<0,001	0,582
**36:2**	3,0 ± 0,8	3,5 ± 1,2	2,6 ± 0,4	0,713	0,674	0,444
**36:3**	2,6 ± 0,6	1,3 ± 0,4	0,8 ± 0,1	0,141	0,017	0,217
**36:4**	1,3 ± 0,2 a	0,5 ± 0,2	0,2 ± 0,0	0,059	0,007	0,172
**TG (16:0)**						
**32:1**	4,2 ± 0,4	9,2 ± 2,3	4,6 ± 0,6	0,163	0,633	0,048
**32:2**	2,3 ± 0,6	1,9 ± 0,6	1,3 ± 0,2	0,673	0,126	0,298
**32:3**	0,3 ± 0,1	0,4 ± 0,1	0,2 ± 0,1	0,9	0,423	0,482
**34:1**	16,7 ± 1,9	26,7 ± 9,9	19,9 ± 3,3	0,438	0,524	0,495
**34:2**	27,1 ± 5,0	17,2 ± 6,3	11,5 ± 1,5	0,266	0,013	0,355
**34:3**	8,1 ± 1,9	3,2 ± 1,0	2,0 ± 0,2	0,062	0,008	0,258
**34:4**	0,9 ± 0,2	0,3 ± 0,1	0,2 ± 0,0	0,052	0,019	0,327
**35:2**	1,1 ± 0,3	2,5 ± 1,0	1,8 ± 0,2	0,22	0,073	0,488
**35:3**	0,5 ± 0,1	0,6 ± 0,2	0,4 ± 0,0	0,61	0,48	0,299
**36:2**	51,5 ± 12,3	64,9 ± 23,2	47,0 ± 5,0	0,628	0,723	0,424
**36:3**	52,1 ± 9,2	24,6 ± 6,8	16,5 ± 2,2	0,053	0,004	0,249
**36:4**	28,1 ± 3,9 a	4,7 ± 1,2	2,9 ± 0,4	0,001	<0,001	0,14
**36:5**	5,6 ± 0,7 a	0,7 ± 0,1	0,4 ± 0,1	<0,001	<0,001	0,073
**37:3**	0,4 ± 0,1	0,2 ± 0,0 a	0,2 ± 0,0	0,545	0,048	0,566
**38:2**	0,9 ± 0,2	1,0 ± 0,4	0,8 ± 0,1	0,79	0,783	0,612
**38:3**	1,4 ± 0,4	0,9 ± 0,4	0,7 ± 0,1	0,458	0,091	0,532
**38:4**	2,1 ± 0,6	0,7 ± 0,1	1,0 ± 0,1	0,112	0,08	0,286
**38:5**	2,8 ± 0,6	1,1 ± 0,3	0,7 ± ,1	0,057	0,009	0,238
**38:6**	2,0 ± 0,4	0,7 ± 0,1	0,5 ± 0,1	0,024	0,006	0,219
**40:6**	1,4 ± 0,3	0,8 ± 0,2	0,6 ± 0,1	0,161	0,021	0,269
**40:7**	2,5 ± 0,5	1,3 ± 0,3	0,9 ± 0,1 a	0,096	0,022	0,177
**40:8**	1,3 ± 0,2 a	0,3 ± 0,1	0,2 ± 0,0 a	0,003	0,005	0,163
**TG (16:1)**						
**34:0**	1,5 ± 0,3	2,0 ± 0,8	1,4 ± 0,1	0,626	0,75	0,437
**34:1**	11,3 ± 2,8	10,7 ± 4,2	7,4 ± 1,0	0,91	0,199	0,423
**34:2**	10,3 ± 2,8	4,7 ± 1,7	3,1 ± 0,3	0,143	0,023	0,324
**34:3**	2,3 ± 0,7	0,7 ± 0,2	0,4 ± 0,0	0,06	0,02	0,133
**36:1**	2,3 ± 0,8	3,0 ± 1,1	2,3 ± 0,3	0,657	0,928	0,507
**36:2**	15,2 ± 5,3	6,8 ± 0,8	8,5 ± 0,7	0,243	0,202	0,168
**36:3**	11,1 ± 3,0	3,5 ± 1,0	2,6 ± 0,2	0,052	0,015	0,33
**36:4**	3,9 ± 0,8 a	0,7 ± 0,2	0,5 ± 0,1	0,007	0,002	0,23
**38:3**	0,5 ± 0,1	0,3 ± 0,1	0,2 ± 0,0 b	0,387	0,061	0,191
**38:4**	0,7 ± 0,3	0,2 ± 0,0	0,2 ± 0,0 a	0,185	0,084	0,83
**38:5**	1,0 ± 0,2	0,3 ± 0,1	0,2 ± 0,0	0,054	0,017	0,662

**TG (17:0)**						
**34:2**	0,5 ± 0,1	1,1 ± 0,4	0,6 ± 0,1	0,232	0,503	0,249
**36:3**	0,7 ± 0,2	1,0 ± 0,3	0,8 ± 0,1	0,347	0,646	0,369
**36:4**	0,6 ± 0,1 a	0,3 ± 0,1	0,2 ± 0,0	0,077	0,001	0,313
**TG (17:1)**						
**34:1**	0,7 ± 0,1	2,3 ± 1,1	1,6 ± 0,2	0,284	0,026	0,473
**34:2**	0,6 ± 0,2	0,9 ± 0,3	0,6 ± 0,1	0,572	0,834	0,371
**36:3**	0,4 ± 0,0	0,5 ± 0,2	0,4 ± 0,0	0,431	0,914	0,307
**TG (18:0)**						
**32:2**	0,4 ± 0,1	0,9 ± 0,3	0,5 ± 0,0	0,168	0,122	0,229
**34:2**	3,3 ± 0,1	5,0 ± 1,6	2,9 ± 0,3	0,423	0,397	0,197
**34:3**	1,1 ± 0,2	0,9 ± 0,3	0,6 ± 0,0	0,595	0,067	0,321
**36:3**	8,2 ± 2,0	4,8 ± 1,2	3,5 ± 0,2	0,192	0,03	0,247
**36:4**	7,3 ± 1,4 a	1,2 ± 0,3	0,8 ± 0,1	0,005	0,001	0,192
**38:6**	0,5 ± 0,1 a	0,3 ± 0,0	0,3 ± 0,0	0,054	0,019	0,5
**TG (18:1)**						
**30:0**	2,5 ± 0,0	3,8 ± 1,1	3,0 ± 0,4	0,461	0,509	0,461
**30:1**	1,5 ± 0,1	2,4 ± 0,9	1,5 ± 0,2	0,403	0,775	0,314
**32:0**	7,5 ± 1,4	15,6 ± 5,6	11,4 ± 1,8	0,206	0,151	0,447
**32:1**	15,1 ± 4,0	16,4 ± 6,5	11,9 ± 1,5	0,877	0,429	0,474
**32:2**	4,9 ± 1,2	3,2 ± 1,1	2,3 ± 0,2	0,338	0,053	0,369
**32:3**	0,5 ± 0,1 a	0,3 ± 0,0	0,3 ± 0,0	0,082	0,039	0,952
**33:2**	0,8 ± 0,2	0,7 ± 0,1	0,8 ± 0,1	0,732	0,919	0,285
**34:1**	82,1 ± 22,8	108,6 ± 39,4	84,2 ± 10,8	0,582	0,931	0,528
**34:2**	71,2 ± 17,2	37,6 ± 11,6	28,0 ± 3,2	0,158	0,028	0,403
**34:3**	14,4 ± 3,6	5,5 ± 1,5	3,7 ± 0,4	0,065	0,013	0,248
**34:4**	1,6 ± 0,3 a	0,6 ± 0,2	0,4 ± 0,0	0,042	0,004	0,113
**35:2**	2,2 ± 0,7	3,4 ± 1,2	2,9 ± 0,1	0,459	0,261	0,715
**35:3**	0,7 ± 0,2	0,7 ± 0,2	0,5 ± 0,0	0,85	0,231	0,444
**36:3**	74,8 ± 22,6	16,0 ± 0,0	16,4 ± 1,7	0,157	0,042	0,86
**36:4**	39,4 ± 8,4 a	5,2 ± 1,2	3,3 ± 0,2	0,019	0,005	0,117
**36:5**	8,7 ± 1,8 a	0,9 ± 0,2	0,6 ± 0,1	0,004	0,001	0,203
**38:5**	5,0 ± 1,8	1,6 ± 0,3	1,2 ± 0,1	0,113	0,048	0,244
**38:6**	3,9 ± 0,9	1,5 ± 0,3	1,2 ± 0,2	0,05	0,015	0,333
**38:7**	0,9 ± 0,2 a	0,4 ± 0,1	0,4 ± 0,0	0,025	0,054	0,709
**TG (18:2)**						
**30:0**	1,2 ± 0,2	1,0 ± 0,3	0,6 ± 0,0	0,625	0,007	0,151
**30:1**	0,7 ± 0,1	0,4 ± 0,1	0,3 ± 0,0	0,157	0,022	0,2
**32:0**	8,7 ± 1,6	4,0 ± 1,1	2,5 ± 0,3	0,047	0,003	0,179
**32:1**	7,7 ± 1,6	3,2 ± 1,0	2,2 ± 0,2	0,05	0,005	0,269
**32:2**	2,9 ± 0,5 a	0,6 ± 0,2	0,4 ± 0,0	0,006	0,003	0,326
**33:0**	0,5 ± 0,1	0,7 ± 0,3	0,4 ± 0,1	0,422	0,89	0,329
**33:1**	0,9 ± 0,2	1,0 ± 0,3	0,7 ± 0,1	0,941	0,203	0,437
**34:0**	6,9 ± 1,2	4,5 ± 1,3	3,1 ± 0,3	0,234	0,013	0,285
**34:1**	46,1 ± 7,9	22,2 ± 6,5	15,2 ± 1,9	0,057	0,004	0,287
**34:2**	47,9 ± 8,3 a	6,8 ± 1,5	4,6 ± 0,7	0,003	<0,001	0,191
**34:3**	8,9 ± 1,5 a	1,0 ± 0,3	0,6 ± 0,1	0,002	<0,001	0,191
**35:1**	0,9 ± 0,2	1,0 ± 0,2	0,8 ± 0,1	0,803	0,55	0,447
**35:2**	1,2 ± 0,3	0,6 ± 0,1	0,5 ± 0,1	0,153	0,059	0,568
**36:0**	1,1 ± 0,3	1,0 ± 0,3	0,7 ± 0,1	0,836	0,127	0,232
**36:1**	10,6 ± 2,7	5,1 ± 1,3	3,6 ± 0,4	0,117	0,024	0,256

**36:2**	45,0 ± 12,0	11,1 ± 2,8	8,4 ± 0,8	0,065	0,023	0,337
**36:3**	54,4 ± 10,8 a	3,8 ± 0,6	2,6 ± 0,2	0,011	0,003	0,089
**38:4**	2,1 ± 0,6	0,4 ± 0,1	0,3 ± 0,0	0,033	0,014	0,385
**38:5**	2,7 ± 0,5 a	0,4 ± 0,1	0,3 ± 0,0	0,006	0,002	0,088
**38:6**	2,5 ± 0,5 a	0,3 ± 0,1	0,2 ± 0,0 a	0,004	0,003	0,275
**TG (18:3)**						
**32:0**	1,2 ± 0,2	0,6 ± 0,2	0,4 ± 0,0 a	0,038	<0,001	0,276
**32:1**	0,9 ± 0,2	0,5 ± 0,1	0,3 ± 0,0	0,069	0,011	0,111
**34:0**	1,0 ± 0,2 a	0,6 ± 0,2	0,4 ± 0,1	0,135	0,006	0,262
**34:1**	6,8 ± 1,0 a	2,9 ± 0,8	1,8 ± 0,2	0,022	<0,001	0,161
**34:2**	6,9 ± 1,0 b	1,0 ± 0,3	0,6 ± 0,1	0,001	<0,001	0,203
**36:1**	1,6 ± 0,4 a	0,7 ± 0,2	0,6 ± 0,1	0,056	0,028	0,522
**36:2**	7,1 ± 1,6 a	1,6 ± 0,4	1,0 ± 0,1	0,017	0,004	0,183
**36:3**	8,4 ± 1,6 a	0,5 ± 0,1	0,3 ± 0,1	0,003	<0,001	0,135
**TG (20:1)**						
**34:1**	0,7 ± 0,2	1,1 ± 0,5	0,8 ± 0,1	0,541	0,83	0,546
**34:2**	0,9 ± 0,2	0,5 ± 0,2	0,3 ± 0,0	0,165	0,024	0,241
**TG (20:2)**						
**34:1**	0,8 ± 0,2	0,3 ± 0,0	0,5 ± 0,1	0,14	0,193	0,101
**TG (20:3)**						
**32:1**	0,5 ± 0,0	0,3 ± 0,1	0,2 ± 0,0	0,273	0,008	0,732
**34:0**	0,3 ± 0,1	0,4 ± 0,1	0,3 ± 0,0	0,797	0,814	0,585
**34:1**	2,5 ± 0,8	2,2 ± 0,9	1,7 ± 0,2	0,264	0,368	0,09
**34:2**	2,0 ± 0,6	1,0 ± 0,3	0,6 ± 0,0	0,102	0,033	0,873
**36:3**	2,0 ± 0,7	0,6 ± 0,1	0,4 ± 0,0	0,068	0,025	0,138
**36:4**	0,8 ± 0,2 a	0,2 ± 0,0	0,2 ± 0,0	0,035	0,078	0,324
**TG (20:4)**						
**32:0**	0,8 ± 0,1	0,5 ± 0,1	0,4 ± 0,0	0,048	0,006	0,357
**32:1**	0,9 ± 0,2	0,4 ± 0,1	0,3 ± 0,0	0,09	0,018	0,511
**34:0**	0,9 ± 0,2	0,6 ± 0,1	0,5 ± 0,1	0,39	0,12	0,384
**34:1**	6,5 ± 1,5	2,7 ± 0,6	2,0 ± 0,2	0,056	0,012	0,294
**34:2**	6,2 ± 1,3	1,1 ± 0,2	0,8 ± 0,1	0,009	0,003	0,273
**34:3**	1,2 ± 0,2 a	0,2 ± 0,0	0,2 ± 0,0	0,01	0,01	0,674
**36:2**	7,3 ± 2,2	1,8 ± 0,2	1,5 ± 0,2	0,05	0,022	0,3
**36:3**	6,2 ± 1,6 a	0,7 ± 0,1	0,6 ± 0,1	0,015	0,006	0,19
**36:4**	3,3 ± 0,7	0,3 ± 0,1	0,2 ± 0,0	0,006	0,006	0,117
**TG (20:5)**						
**34:1**	1,3 ± 0,2 a	1,0 ± 0,2	0,6 ± 0,1	0,331	0,013	0,059
**34:2**	1,1 ± 0,1 b	0,5 ± 0,1	0,3 ± 0,0	0,007	0,002	0,112
**36:2**	1,3 ± 0,4 a	0,6 ± 0,1	0,4 ± 0,0	0,111	0,043	0,104
**36:3**	1,1 ± 0,2 b	0,3 ± 0,0	0,2 ± 0,0	0,009	0,002	0,039
**TG (22:5)**						
**34:1**	2,3 ± 0,6	1,3 ± 0,3	0,8 ± 0,1	0,186	0,033	0,166
**34:2**	1,5 ± 0,4	0,4 ± 0,1	0,3 ± 0,0	0,032	0,008	0,172
**TG (22:6)**						
**32:0**	1,1 ± 0,2	0,8 ± 0,1	0,6 ± 0,2	0,262	0,088	0,404
**32:1**	1,3 ± 0,3	0,6 ± 0,1	0,5 ± 0,1	0,06	0,016	0,458
**34:1**	8,4 ± 1,7	5,1 ± 1,0	3,5 ± 0,2 a	0,15	0,033	0,188
**34:2**	7,5 ± 1,3 a	1,9 ± 0,4	1,0 ± 0,0 a	0,005	0,002	0,061

**34:3**	1,3 ± 0,2 b	0,3 ± 0,1	0,2 + 0,0	0,002	<0,001	0,472

CONV – Glycosylceramide

Metabolite	SCD	HFD	HFD + Ator	p: SCD : HFD	p: SCD : HFD + Ator	p: HFD : HFD + Ator
**HexCer**						
**(d18:1/16:0)**	1,0 ± 0,1	1,4 ± 0,1	1,2 ± 0,1	0,041	0,076	0,297
**(d18:1/18:0)**	0,3 ± 0,0	0,7 ± 0,1	0,5 ± 0,1	0,003	0,074	0,177
**(d18:1/18:1)**	0,0 ± 0,0	0,1 ± 0,0	0,1 ± 0,0	0,002	0,022	0,023
**(d18:1/20:0)**	0,2 ± 0,0	0,5 ± 0,1	0,4 ± 0,1	0,002	0,081	0,069
**(d18:1/22:0)**	2,0 ± 0,0	3,0 ± 0,3	2,5 ± 0,3	0,046	0,223	0,24
**(d18:1/23:0)**	0,7 ± 0,1	1,8 ± 0,2	1,3 ± 0,1	<0,001	0,012	0,025
**(d18:1/24:0)**	0,5 ± 0,0	0,7 ± 0,1	0,6 ± 0,0	0,017	0,101	0,15
**(d18:1/24:1)**	3,2 ± 0,2	3,4 ± 0,3	2,9 ± 0,2	0,558	0,333	0,187

ABS – Glycosylceramide

Metabolite	SCD	HFD	HFD + Ator	p: SCD : HFD	p: SCD : HFD + Ator	p: HFD : HFD + Ator
**HexCer**						
**(d18:1/16:0)**	0,5 ± 0,1 b	0,8± 0,0 b	0,8 ± 0,0 b	0,034	0,031	0,737
**(d18:1/18:0)**	0,2 ± 0,0 a	0,4 ± 0,0 a	0,3 ± 0,0	0,002	0,011	0,11
**(d18:1/18:1)**	0,0 ± 0,0 a	0,1 ± 0,0	0,0 ± 0,0	0,048	0,55	0,147
**(d18:1/20:0)**	0,2 ± 0,0	0,4 ± 0,0 a	0,3 ± 0,0	0,063	0,096	0,348
**(d18:1/22:0)**	1,2 ± 0,2 b	1,9 ± 0,2 a	1,6 ± 0,1 a	0,025	0,056	0,154
**(d18:1/23:0)**	0,6 ± 0,1	1,0 ± 0,0 b	0,9 ± 0,0 a	0,011	0,01	0,5
**(d18:1/24:0)**	0,4 ± 0,1	0,5 ± 0,0 a	0,4 ± 0,0 a	0,174	0,391	0,324
**(d18:1/24:1)**	2,2 ± 0,4	2,0 ± 0,1 b	1,7 ± 0,1 b	0,549	0,17	0,1

Supplemental Table S3

Table summarizes the of effect of atorvastatin on selected lipids including glycerophospholipids, triacylglycerols and glycosylceramide in conventionally raised (CONV) and abiotic mice (ABS). Significantly increased values in ABS mice as compared to the respective group in CONV mice are highlighted in green and the significantly decreased values are highlighted in red, respectively. The letters a,b and c indicate the degree of significance: with a = p<0.05; b = p<0.01; and c = p<0.001.

References

1. Lee, D.H.; Zo, Y.G.; Kim, S.J. Nonradioactive method to study genetic profiles of natural bacterial communities by PCR-single-strand-conformation polymorphism. *Appl. Environ. Microbiol.* **1996**, *62*, 3112–3120, doi:10.1128/aem.62.9.3112-3120.1996.
2. Van Dyke, M.I.; McCarthy, A.J. Molecular Biological Detection and Characterization of Clostridium Populations in Municipal Landfill Sites. *Appl. Environ. Microbiol.* **2002**, *68*, 2049–2053, doi:10.1128/aem.68.4.2049-2053.2002.
3. Matsuki, T.; Watanabe, K.; Fujimoto, J.; Miyamoto, Y.; Takada, T.; Matsumoto, K.; Oyaizu, H.; Tanaka, R. Development of 16S rRNA-Gene-Targeted Group-Specific Primers for the Detection and Identification of Predominant Bacteria in Human Feces. *Appl. Environ. Microbiol.* **2002**, *68*, 5445–5451, doi:10.1128/aem.68.11.5445-5451.2002.
4. Bartosch, S.; Fite, A.; Macfarlane, G.T.; McMurdo, M.E.T. Characterization of Bacterial Communities in Feces from Healthy Elderly Volunteers and Hospitalized Elderly Patients by Using Real-Time PCR and Effects of Antibiotic Treatment on the Fecal Microbiota. *Appl. Environ. Microbiol.* **2004**, *70*, 3575–3581, doi:10.1128/aem.70.6.3575-3581.2004.
5. Kühbacher, T.; Ott, S.J.; Helwig, U.; Mimura, T.; Rizzello, F.; Kleessen, B.; Gionchetti, P.; Blaut, M.; Campieri, M.; Fölsch, U.R.; et al. Bacterial and fungal microbiota in relation to probiotic therapy (VSL#3) in pouchitis. *Gut* **2006**, *55*, 833–841, doi:10.1136/gut.2005.078303.
6. Heilig, H.G.; Zoetendal, E.G.; Vaughan, E.E.; Marteau, P.; Akkermans, A.D.; De Vos, W.M. Molecular Diversity of Lactobacillus spp. and Other Lactic Acid Bacteria in the Human Intestine as Determined by Specific Amplification of 16S Ribosomal DNA. *Appl. Environ. Microbiol.* **2002**, *68*, 114–123, doi:10.1128/aem.68.1.114-123.2002.
7. Walter, J.; Hertel, C.; Tannock, G.W.; Lis, C.M.; Munro, K.; Hammes, W.P. Detection of Lactobacillus, Pediococcus, Leuconostoc, and Weissella Species in Human Feces by Using Group-Specific PCR Primers and Denaturing Gradient Gel Electrophoresis. *Appl. Environ. Microbiol.* **2001**, *67*, 2578–2585, doi:10.1128/AEM.67.6.2578-2585.2001.
8. Rinttilä, T.; Kassinen, A.; Malinen, E.; Krogius, L.; Palva, A. Development of an extensive set of 16S rDNA-targeted primers for quantification of pathogenic and indigenous bacteria in faecal samples by real-time PCR. *J. Appl. Microbiol.* **2004**, *97*, 1166–1177, doi:10.1111/j.1365-2672.2004.02409.x.
9. Barman, M.; Unold, D.; Shifley, K.; Amir, E.; Hung, K.; Bos, N.; Salzman, N. Enteric Salmonellosis Disrupts the Microbial Ecology of the Murine Gastrointestinal Tract. *Infect. Immun.* **2007**, *76*, 907–915, doi:10.1128/iai.01432-07.
10. Wise, M.; Siragusa, G.R. Quantitative analysis of the intestinal bacterial community in one- to three-week-old commercially reared broiler chickens fed conventional or antibiotic-free vegetable-based diets. *J. Appl. Microbiol.* **2006**, *102*, 1138–1149, doi:10.1111/j.1365-2672.2006.03153.x.
